# Eliciting an immune-mediated antitumor response through oncolytic herpes simplex virus-based shared antigen expression in tumors resistant to viroimmunotherapy

**DOI:** 10.1136/jitc-2021-002939

**Published:** 2021-10-01

**Authors:** Mohammed G Ghonime, Uksha Saini, Michael C Kelly, Justin C Roth, Pin-Yi Wang, Chun-Yu Chen, Katherine Miller, Ilse Hernandez-Aguirre, Yeaseul Kim, Xiaokui Mo, Joseph R Stanek, Tim Cripe, Elaine Mardis, Kevin A Cassady

**Affiliations:** 1 Center for Childhood Cancer and Blood Disorders, Abigail Wexner Research Institute at Nationwide Children's Hospital, Columbus, Ohio, USA; 2 The University of Alabama at Birmingham School of Medicine, Birmingham, Alabama, USA; 3 The Steve and Cindy Rasmussen Institute for Genomic Medicine, Abigail Wexner Research Institute at Nationwide Children's Hospital, Columbus, Ohio, USA; 4 College of Medicine, The Ohio State University, Columbus, Ohio, USA; 5 Biostatistics, The Ohio State University, Columbus, Ohio, USA; 6 Biostatistics Resource, Nationwide Children's Hospital, Columbus, Ohio, USA; 7 Pediatrics, The Ohio State University, Columbus, Ohio, USA; 8 The Steve and Cindy Rasmussen Institute for Genomic Medicine, Nationwide Children's Hospital, Columbus, Ohio, USA

**Keywords:** immunotherapy, oncolytic virotherapy, brain neoplasms, sarcoma, immunological memory

## Abstract

**Background:**

Oncolytic virotherapy (OV) is an immunotherapy that incorporates viral cancer cell lysis with engagement of the recruited immune response against cancer cells. Pediatric solid tumors are challenging targets because they contain both an inert immune environment and a quiet antigenic landscape, making them more resistant to conventional OV approaches. Further complicating this, herpes simplex virus suppresses host gene expression during virotherapy infection.

**Methods:**

We therefore developed a multimodal oncolytic herpes simplex virus (oHSV) that expresses ephrin A2 (EphA2), a shared tumor-associated antigen (TAA) expressed by many tumors to improve immune-mediated antitumor activity. We verified the virus genotypically and phenotypically and then tested it in an oHSV-resistant orthotopic model (including immunophenotypic analysis), in flank and in T cell-deficient mouse models. We then assessed the antigen-expressing virus in an unrelated peripheral tumor model that also expresses the shared tumor antigen and evaluated functional T-cell response from the treated mice.

**Results:**

Virus-based EphA2 expression induces a robust acquired antitumor immune responses in both an oHSV-resistant murine brain and peripheral tumor model. Our new multimodal oncolytic virus (1) improves survival in viroimmunotherapy resistant tumors, (2) alters both the infiltrating and peripheral T-cell populations capable of suppressing tumor growth on rechallenge, and (3) produces EphA2-specific CD8 effector-like populations.

**Conclusions:**

Our results suggest that this flexible viral-based platform enables immune recognition of the shared TAA and improves the immune-therapeutic response, thus making it well suited for low-mutational load tumors.

## Introduction

Therapeutic advancements have improved the outcome for many cancers; however, some tumors remain resistant to conventional strategies and require different therapeutic approaches. Cancer immunotherapy is a novel treatment option that involves priming the immune system for tumor cell eradication. Immunotherapies have been used to overcome the limited efficacy of the classic therapeutic options like surgery, radiotherapy, chemotherapy, and therapy for patients with advanced stage solid tumor.^1^ However, not all cancers are responsive to immunotherapy. A paucity of lymphocytic immune cell infiltrates and a metabolically restrictive immunosuppressive microenvironment can limit the efficacy of this approach.^2^ In other cases, limited neoantigen targets due to low mutational loads have also been proposed to limit durable antitumor immunotherapeutic responses.^1^

Malignant gliomas (MGs) and malignant peripheral nerve sheath tumors (MPNSTs) are two highly treatment-resistant cancers that affect adults and children and involve the nervous system.^3–5^ Gliomas are highly vascular and complex tumors containing both paradoxically inflamed and immune-resistant environments.^6^ Surgical resection followed by temozolomide and radiation therapy has improved median survival; however, most patients suffer local regrowth of tumor and median survival remains <2 years for most patients. MPNSTs are solid tumors of the peripheral nervous system with a complex tumor biology and equally poor prognosis.^3 7^ MPNSTs are resistant to conventional therapies (chemotherapy and radiation therapy), and surgery remains the mainstay of treatment.^7^ Unfortunately, these tumors have high local recurrence rates and, at the time of tumor discovery, metastatic disease is present in the majority of patients, leading to a 5-year survival rate between 23% and 69%.^7 8^

Cancer cells often express tumor-associated antigens (TAAs) that not only contribute to malignant transformation, tumor cell survival, and metastases but also can serve as immunotherapeutic targets.^9–11^ These ‘shared’ proteins (eg, ephrin A2 (EphA2), interleukin 13 receptor alpha 2, survivin (BIRC5), and epidermal growth factor receptor variant 3) are often expressed in non-malignant tissue during periods of high mitotic activity (eg, embryogenesis) or in immune-privileged sites but can also be aberrantly expressed in tumors (overexpression and different subcellular localization), where they provide a survival or growth advantage for malignant cells.^12–15^ They are difficult to generate immune activity because the immune system recognizes these antigens as self-proteins, and high-affinity T cells are eliminated by clonal deletion as part of central tolerance.^16^

EphA2 is one such shared antigen that belongs to the Eph subfamily of receptor tyrosine kinases. The protein contributes to cellular proliferation, migration, repulsion, and adhesion during neuronal, vascular, and epithelial development, and is expressed by immune cells and osteoclasts where it participates in bone remodeling.^17^ EphA2 is also often upregulated in some mesenchymal tumors (melanoma and MGs) and in epithelial cancers (ovarian, cervical, prostate, breast, gastric, lung, colon, esophageal, and bladder cancers).^18–20^ As a tumor antigen, EphA2 is associated with tumor initiation, angiogenesis, metastasis, and overall poor prognosis.^21^ Ephrin family members use a complex bidirectional signaling mechanism where it can act both as a ligand and receptor.^22^ Because of its expression in a broad and large number of cancers, Epha2 has also been targeted therapeutically.^9 20^ Because of its high functional complexity, EphA2 can produce ‘ligand-independent’ stimulatory activity in some tumors and, in others, generate a ligand-dependent tumor-suppressive effect.^23 24^ Therefore, investigators have used both direct EphA2 inhibition (small molecule inhibitors, ephrin receptor blocking peptides, or antibodies) to block stimulatory kinase signaling as well as transient EphA2 pathway stimulation to induce inhibitory pathways and feedback responses to reduce tumor growth through this pathway.^20^ For our studies, we sought to target this ubiquitous TAA and improve the immunotherapeutic response against the tumor using virotherapy.

Viroimmunotherapy uses viruses (both naturally occurring or genetically modified agents) to preferentially target tumor cells, inducing both a direct cytolytic as well as an immune-mediated lytic response against the cancer.^4 25^ Oncolytic herpes simplex virus (oHSV) is one of many virus-based platforms and has been safely used in clinical trials for a wide range of cancer types, including brain tumors.^4^ Although traditional oncolytic virotherapy (OV) approaches can promote neoantigen recognition by adaptive immune cells, this process is less likely to be effective in low-mutational load tumors.^25^ Therefore, we postulated that OV-based immunostimulation and expression of shared tumor antigens would be an effective approach to prime immune response in a low-mutational load tumor.^25^ Viral infections are well described at breaking immune tolerance and inducing autoimmunity to self-antigens.^26 27^ Herpes simplex virus (HSV) is no exception and, in some patients, has been shown to induce a systemic autoimmune reaction (erythema multiforme), a T cell-mediated keratitis, or an autoimmune-mediated encephalitis.^28–30^ Like other viruses, HSV has evolved mechanisms to survive the antiviral response and confines the peak of the immune response during its lytic phase, but over time, immunity and local inflammation suppress lytic viral infection.^31^ Nevertheless, HSV produces a lifelong infection in humans, periodically reactivating from its latent form. During HSV reactivation, the virus can replicate despite prior immunity, although the duration of lytic infection is shortened by a memory population that responds to the virus-infected cells.^32^

While oHSV infection recruits immune effectors, it also suppresses host gene expression in the infected cell.^33–35^ This enables selective viral gene expression but also likely suppresses immune recognition of host genes, including tumor antigens, during oHSV infection.^36^ We hypothesized that OV-based expression of a shared TAA would enhance TAA immune recognition and improve OV antitumor activity. To test this hypothesis, we used a next-generation oHSV (C134) to create recombinants that encode the full length and secreted murine (C57BL/6) EphA2. Using two different C57BL/6-based immune-competent tumor models, our results show that oHSV-based tumor antigen expression improves survival and antitumor activity in two oHSV-resistant tumor models. Virus-based tumor antigen expression not only induces tumor-infiltrating leukocyte (TIL) and peripheral immune cell phenotypical changes but also produces functional populations with an improved Epha2 antigen response capable of suppressing tumor growth on rechallenge of survivors. Our exciting findings confirm that OVs can be modified to enhance immune recognition of ‘self’ TAAs and that this strategy of in situ antitumor vaccination can be harnessed as a virotherapeutic approach in the low-mutational load environment. Taken together, our results suggest that this therapeutic approach is not limited to one unique animal model and may be applicable to other EphA2(+) tumors beyond nervous system tumors (eg, triple negative breast cancer, pancreatic, ovarian, colon, prostate, sarcoma, and MGs).

## Materials and methods

### Cell lines and viruses

CT2A cells were kindly provided by Dr Thomas Seyfried (Boston College, Chestnut Hill, Massachusetts, USA) and were propagated in Dulbecco’s modified eagle medium (DMEM) supplemented with 10% fetal bovine serum (FBS). 67 C-4 was kindly provided by Dr Nancy Ratner (University of Cincinnati, Cincinnati, Ohio, USA) and maintained in DMEM supplemented with 10% FBS. Vero cells (ATCC, Manassas, Virginia, USA) were used for growing virus selection, stock preparations, and limiting plaque dilution titration studies. Tumor lines were tested negative for mycoplasma contamination by PCR and in vivo detect (Invivogen, San Diego, California, USA). Tumor cells with relatively low passage numbers (<12 passages) were used in the study before returning for a ‘low’ passage form of the cell line to minimize genetic drift in our studies. The HSV-1 (F) strain was kindly provided by Dr Bernard Roizman (University of Chicago, Chicago, Illinois, USA); C134 has been described previously.^37 38^ Briefly, C134 is a Δγ_1_34.5 virus that contains the HCMV IRS1 gene under control of the CMV IE promoter in the U_L_3/U_L_4 intergenic region.^38^ C154 is an Enhanced Green Fluorescent Protein (EGFP)-expressing version of C134 with EGFP encoded in the γ_1_34.5 locus. C170 and C172 were created from C154 and encode the MND promoter-driven full length (C170) and extracellularly secreted (C172) C57BL/6 EphA2. Viruses were confirmed genetically by DNA hybridization studies for EphA2 protein expression and cell localization by immunostaining studies from cell lysate and media supernatant samples (western blot), immunocytochemistry of paraformaldehyde fixed and permeabilized cells, and by flow cytometry of non-permeable cell staining.

### Viral spread and recovery assays (in vitro)

CT2A and 67 C-4 cells were plated into clear, 48-well flat-bottom polystyrene tissue culture-treated microplates (Corning, New York, USA) and allowed to adhere overnight at 37°C. Cells were infected the following day with equivalent multiplicity of infection (MOI) of wild-type, C134, and C134-based antigen expressing viruses (C170 and C172), and the plates were monitored using the IncuCyte ZOOM platform, which was housed inside a cell incubator at 37°C with 5% CO_2_ until the end of the assay. Nine images per well from three replicates were taken every 3 hours for 3 days using a ×10 objective lens and then analyzed by phase contrast using the IncuCyte Basic Software. In addition, infected cell samples were harvested at 24-hour intervals postinfection, and viral recovery was measured by limiting plaque dilution similar to that previously described.

### IncuCyte ZOOM viral spread assay

Cells were plated into 96-well flat clear bottom polystyrene tissue culture-treated microplates (Corning) and allowed to adhere overnight. C134 or C170 was added at the indicated MOI, and the plates were transferred into the IncuCyte ZOOM platform, which was housed inside a cell incubator at 37°C with 5% CO_2_, until the end of the assay. Four images per well from three technical replicates were taken every 3 hours for 3 days using a ×10 objective lens and then analyzed using the IncuCyte Basic Software. Green channel acquisition time was 400 ms in addition to phase contrast.

### Western blotting

Cellular lysates from tumor samples were collected on ice in a disruption buffer (10 mM Tris–Cl pH 8.0, 1 mM EDTA, 1% Triton X100, 0.1% sodium deoxycholate, 0.1% Sodium Dodecyl Sulfate (SDS), 140 mM NaCl, 20% β-mercaptoethanol, and 0.04% bromophenol blue) with complete, mini protease inhibitor cocktail (Indianapolis, Indiana, USA). The protein concentrations were determined using Pierce BCA Protein Assay Kit (Thermo Fisher Scientific, Rockford, Illinois, USA). Samples were denatured at 98°C for 5 min, chilled on ice, separated by Polyacrylamide Gel Electrophoresis (PAGE), transferred to a nitrocellulose membrane (Thermo Fisher Scientific) and blocked for 1 hour at room temperature with 5% dry milk (S.T. Jerrell Co.) or bovine serum albumin (Thermo Fisher Scientific). Membranes were incubated overnight at 4°C with primary antibody diluted in Tris-buffered saline with 0.1% Tween-20 (TBST). Primary antibodies against Myc (9B11: 1:1000) were purchased from Cell Signaling Technology. Membranes were repeatedly washed with TBST, incubated for 1 hour with Horseradish Peroxidase (HRP)-conjugated goat antimouse (Pierce) diluted in TBST (1:20,000 dilution) at room temperature and subsequently washed with TBST. Membranes were developed using SuperSignal West Pico Chemiluminescent Substrate (Thermo Fisher Scientific) and exposed to X-ray film (Research Products International).

### Animal tumor studies

Animal studies were approved by the Nationwide Children’s Hospital Institutional Animal Care and Use Committee (IACUC, protocol number AR16-00088 and AR16-00069) and performed in accordance with guidelines established by the National Institutes of Health (NIH) Guide for the Care and Use of Laboratory Animals. Two syngeneic C57BL/6 tumor models were used in these studies: an intracerebral CT2A glioma tumor model and a flank 67 C-4 MPNST model. For the intracerebral studies, C57BL/6 mice 6–8 weeks old were obtained from Envigo (Frederick, Maryland, USA) and were implanted with 1×10^5^ CT2A in 5% methylcellulose using a stereotactic frame, similar to our earlier studies.^37 39^ Five days later, mice were randomized and treated with vehicle or virus (1×10^7^ Plaque Forming Units [PFU]/10 µL) using the same stereotactic coordinates. Mice were assessed daily and moribund mice were sacrificed, and dates were recorded. Mice which survived their CT2A brain tumors also underwent CT2A flank tumor rechallenge to test for circulating functional antitumor response effectors similar to our earlier studies.^39^ Mice were considered long-term responders after no additional tumor-related deaths occurred within a cohort greater than a 1month period. These oHSV-treated ‘survivor mice’ were maintained and, together with a naïve C57BL/6 cohort, were rechallenged with five times the number of tumor cells in their flanks (5×10^5^) and followed up for tumor growth similar to that described previously.

To assess tumor growth characteristics and response to virotherapy, CT2A tumors were also independently implanted in C57BL/6 mice 6–8 weeks old (Envigo) and athymic nude mice (Charles River Laboratory, Wilmington, Massachusetts, USA) by injecting 2×10^6^ CT2A cells in 50 μL of Phosphate Buffered Saline (PBS) /flank. Once tumors were 25–200 mm^3^ in size, mice were randomized and treated with vehicle (10% glycerol in PBS) or 3×10^7^ PFU of C134 or C170 in 50 μL of vehicle. Tumor measurements (length, width, and depth) were performed at least twice per week using digital calipers, and tumor size was calculated.

For MPNST tumor studies, a similar flank tumor approach was used. Murine 67 C-4 MPNST tumor cells (2×10^6^) were injected subcutaneously into the flanks of C57BL/6 mice 6–8 weeks old (Envigo) similar to our earlier studies.^40^ Tumor sizes were measured biweekly by caliper after implantation, and tumor volume was calculated by length×width×depth. When tumors reached 25–150 mm^3^ in size, animals were pooled and randomized into the specified groups, discussed further, with comparable average tumor size. Mice were treated with vehicle, C134 or C170 (3.5×10^7^ PFU in 50 µL 10% glycerol in PBS) intratumorally. Studies were repeated to ensure biological validity. For flank-based studies, animals were monitored for tumor volumes at least two times per week after treatment until an individual tumor was >1500 mm^3^ or in some cases total tumor volume/mouse exceeded 2000 mm^3^. Once tumor size exceeded these criteria, mice were sacrificed based on IACUC requirements.

### Tissue preparation and flow cytometry

For the CT2A brain tumor-based studies, tumor-bearing mice were sacrificed 6 days post-treatment and their brains isolated following saline perfusion as described previously.^14^ In brief, mice following CO_2_ asphyxiation underwent thoracotomy and were perfused with 10–15 cc of sterile normal saline after cannulation of their left ventricle. Mice undergoing saline perfusion exhibited liver blanching, and if this did not occur, the samples were discarded. The isolated brains were placed in Roswell Park Memorial Institute (RPMI) medium and were homogenized by mechanical dissociation using a Qiashredder. The mononuclear cell infiltrate was then isolated from the homogenate by centrifugation over a Percoll 70%/30% step gradient and then underwent immunophenotypical analysis after fluorescent antibody incubation and multiparameter flow cytometry.

Single-cell suspensions from tumors were lysed with Red Blood Cell (RBC) lysis buffer (Sigma, St. Louis, Missouri, USA) and blocked with 5% mouse Fc blocking reagent (2.4G2; BD Biosciences, San Jose, California, USA) in Flow Assisted Cell Sorting (FACS) buffer (1% FBS and 1 mM EDTA in PBS). Cells were labeled with the following antibody staining panels for analysis of the adaptive immune cells: CD11b-Violet 421 (M1/70), CD4-BV785 (GK1.5), CD25-PE (7D4/CD25), CD8a-BV510 (53–6.7), CD3ε-BV 711 (145–2 C11), CD44-APC (IM7), CD45-BV605 (30-F11), NKp46–PE-Cy7 (29A1.4) and B220-AF488 (RA3-6B2), and H2Kb/H2Db (28-8-6) from Bio-Legend (San Diego, California, USA) and mouse EphA2 (REA579) from Miltenyi Biotec (Auburn, California, USA). Dead cells were excluded by staining with live/dead near/Infra Red (IR) staining (APC-Cy7) (Thermo Fisher Scientific, Charlotte, North Carolina, USA). Single samples were stained with the aforementioned staining panels for 30 min on ice and washed one time with FACS buffer. After labeling, cells were fixed in 1% paraformaldehyde and analyzed on a BD FACS LSR II (BD Biosciences). Surface EphA2 and Major Histocompatibility Complex (MHC) I staining were performed using either a BD FACS LSR II (BD Biosciences) or an Attune NxT Acoustic Focusing Cytometer (Thermo Fisher Scientific). Analysis was carried out using FlowJo software V.10.0.3 (Tree Star, Ashland, Oregon, USA) or using Attune NxT Software V.4.2.1627.1 (Thermo Fisher–Life Technologies, Carlsbad, California, USA).

### Peptide pulsing of splenocytes with class I (H2-K^b^)-restricted peptide epitope of EphA2, glycoprotein B, or control peptide

Splenocytes (5×10^5^) from the treated 67C-4 tumor-bearing mice were plated in round-bottom 96-well plates and incubated with and without 10 µM of either EphA2 peptide (671-FSHHNIIRL-679), glycoprotein B peptide (498-SSIEFARL-505), or negative control OVA peptide (SINFFEKL) for 6 hours (similar to our previous studies using identified peptide domains).^40–42^ Samples were incubated with protein transport inhibitor containing 1 µL/mL Brefeldin A (Golgi-plug, BD Biosciences) for 6 hours prior to flow cytometry staining (as described earlier), and CD8 T lymphocytes were analyzed by flow cytometry for surface CD25 (3C7) and intracellular granzyme B (QA16/A02), Interferon (IFN)-γ (XMG1.2), and Tumor Necrosis Factor (TNF)-α (MP6-XT22) staining from Bio-Legend.

### Statistical analysis

Data are summarized by mean±SE. The immune cell population data were analyzed by using one-way analysis of variance (ANOVA) or Kruskal-Wallis test in Prism V.8 (GraphPad Software, San Diego, California, USA) or in SAS9.4. Tumor volumes were first cubic root transformed to ensure normality distribution and then analyzed using mixed effect model by using SAS9.4 software. Survival curves were determined by Kaplan–Meier method and log-rank test was conducted to compare survival between groups using SAS9.4. Studies were repeated at least twice to ensure biological validity. Multiplicity was adjusted by Holm’s procedure to control the type I error rate at 0.05.^43^ For all analyses, the cut-off for statistical significance was set at p<0.05 and the following notation was used: Non Significant (NS)=p>0.05, *p≤0.05, **p≤0.01, ***p≤0.001, ****p≤0.0001.

## Results

### Construction and validation of EphA2 expression viruses

**Figure 1:**
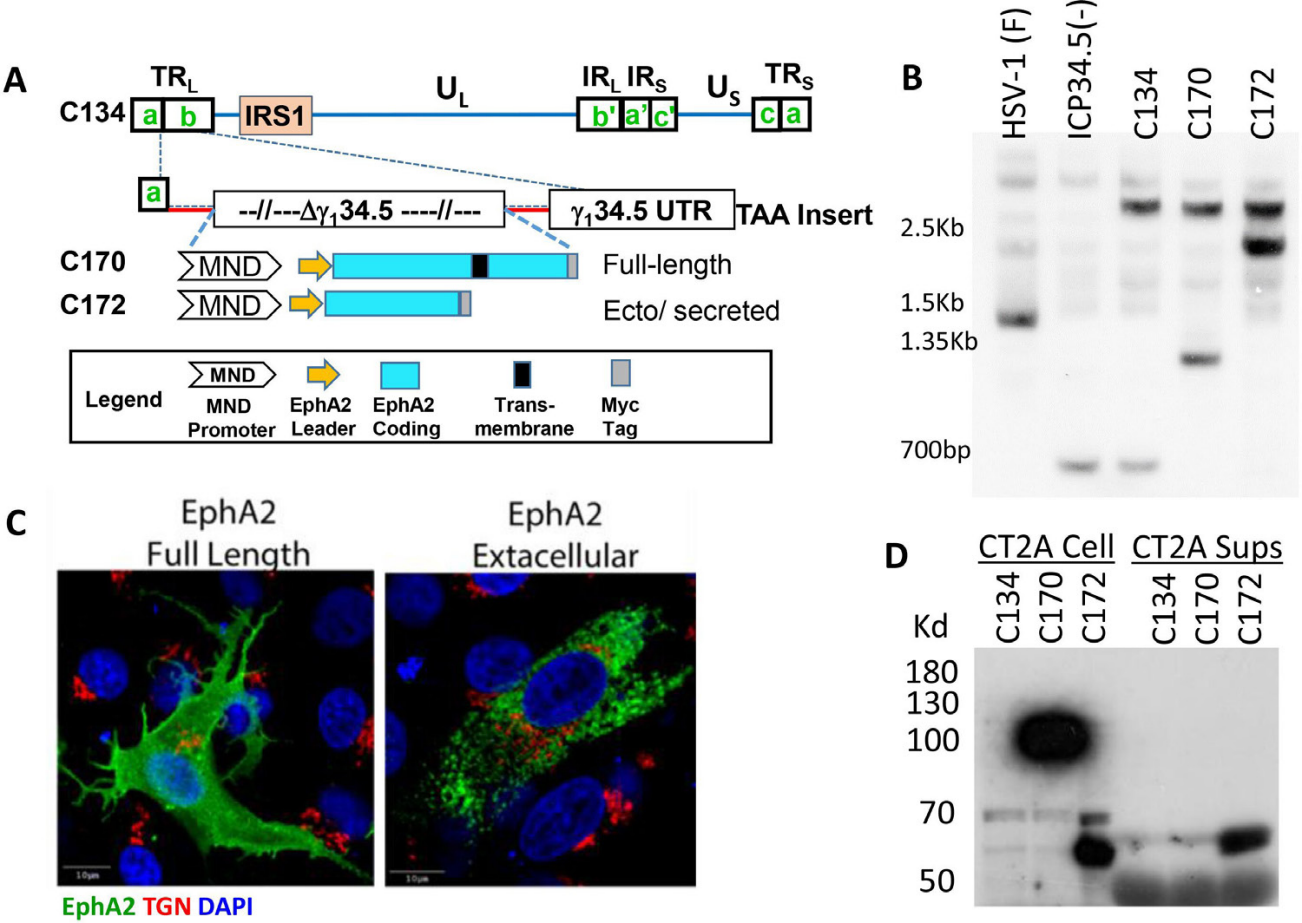
Vector construction, validation and assessment of EphA2 expression. (A) Schematic of the C170 and C172 EphA2 expression viruses. (B) Southern blot confirming the anticipated NcoI fragments in our new recombinants (1.2 and 3.0 kb, C170; 2.3 and 3.0 kb, C172), (C) Immunofluorescent imaging shows different cellular distributions of the full length and secreted forms of the EphA2. (D) Western blots of CT2A infected cells and supernatants show that C170-expressed EphA2 remains cell associated, whereas C172 expressing the extracellular form of EphA2 secretes the protein into the supernatant. TAA: tumor-associated antigen. UTR: untranslated region. TGN: Trans Golgi Network. DAPI: 4′,6-diamidino-2-phenylindole

To test our hypothesis that virus-based TAA expression would produce a tumor antigen-specific antitumor immune response that suppressed tumor growth and improve survival, we created and validated a series of oncolytic HSVs that express regions of the C57BL/6 *EphA2* gene. As summarized in figure 1A, the new recombinants encoded the coding domain of the full length (C170) or secreted extracellular domain of the C57BL/6 *EphA2* sequence in the HSV g_1_34.5 gene locus (C172). The new recombinants were validated by DNA hybridization studies (figure 1B), confocal immunofluorescence (figure 1C) and for cell-associated and secreted protein by western blot (figure 1D). Immunoreactive EphA2 (predicted MW~111.69 kDa) remains cell associated in C170-infected cells (none present in CT2A sups), and the C172 virus produces the smaller but immunoreactive extracellular EphA2 domain (predicted MW 61.17 kDa) that is both cell associated (CT2A cell) and secreted into the CT2A media (CT2A Sups).

### Evaluate direct oncolytic activity in MG tumor model

**Figure 2:**
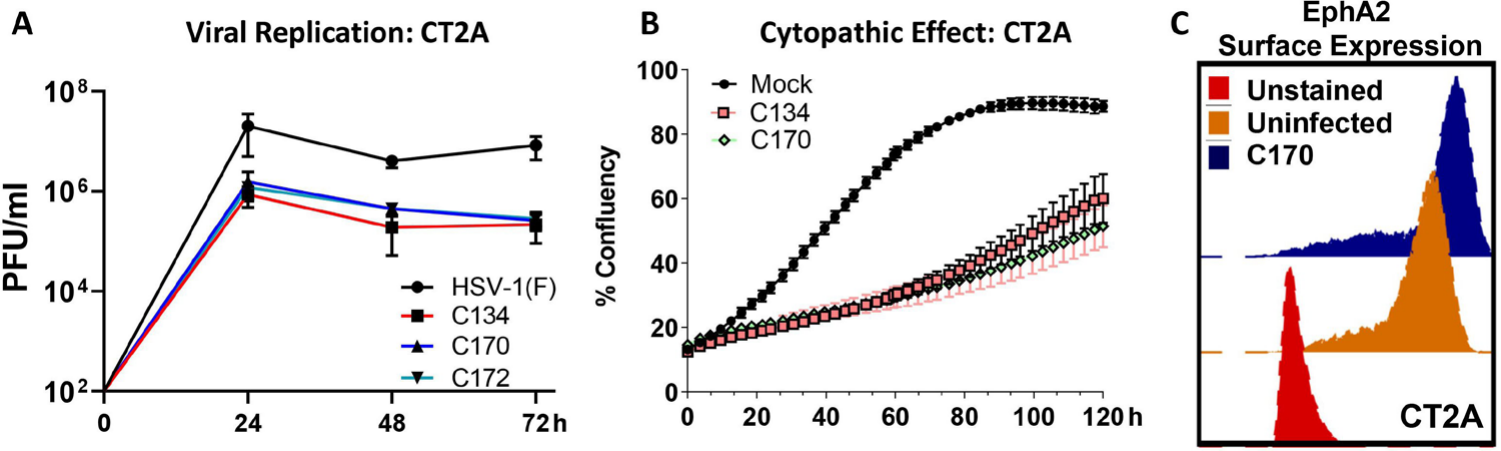
Viral replication and cytopathic effect in C57BL/6 murine CT2A MG panels show viral replication kinetics, cytopathic effect and EphA2 surface expression in infected CT2A C57BL/6-based MG cells. MG, malignant glioma.

Next, we examined the recombinants’ replication or cytopathic changes in our target tumor cells to determine if they had a growth advantage or increased cytolytic activity. The results show that C170 and C172 had a similar replication profile as the parent C134 virus (figure 2A). We also compared tumor cell proliferation and cytopathic effect using IncuCyte cell confluence monitoring. As shown in figure 2B, *Epha2* expression by the virus did not increase CT2A cell proliferation. The results show that C170 and C134 virus produce similar cytopathic activity. To investigate basal and C170 related *EphA2* surface expression in CT2A cells, flow cytometry was also performed. The results show that CT2A cells have high basal *Epha2* expression and that C170 infection further shifted the *EphA2* surface expression based on fluorescent intensity (figure 2C). Additional histograms and calculations of % cells overexpressing EphA2 are provided in online supplemental figure 1.

10.1136/jitc-2021-002939.supp2 Supplementary data

10.1136/jitc-2021-002939.supp1 Supplementary data

### Immunocompetent syngeneic anti -tumor activity

**Figure 3:**
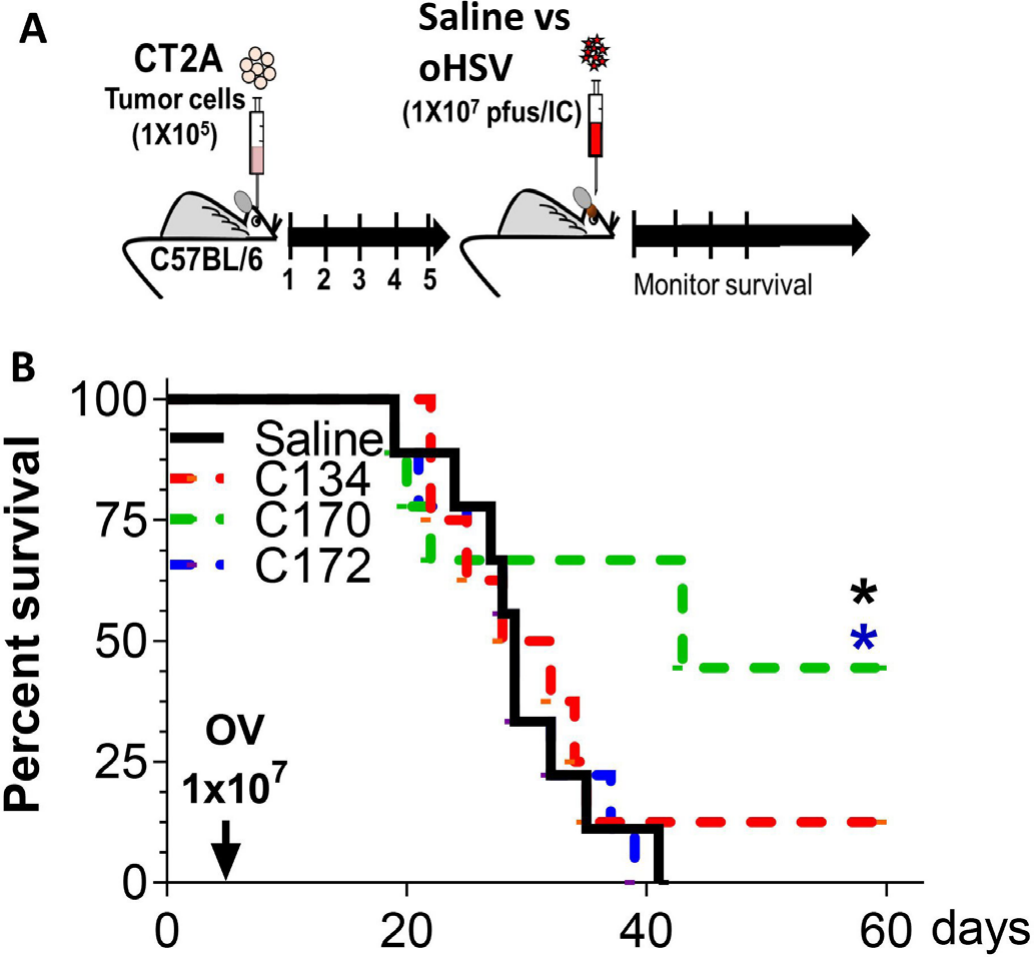
C170 antitumor activity in immunocompetent syngeneic mouse brain tumor model. (A) Schematic representation of study design. (B) Representative survival plot. oHSV, oncolytic herpes simplex virus; OV, oncolytic virotherapy.

To test whether virus-based TAA expression improved antitumor activity and survival, we introduced CT2A brain tumors into B6 mice (1×10^5^ cells) and then treated them 5 days later with saline or equivalent doses of C134, C170, and C172 (1×10^7^ PFU) using the same stereotactic coordinates (summarized in figure 3A). The result show that the CT2A model is oHSV resistant, and neither C134 nor C172 produced significantly improved survival (figure 3B). In contrast, C170 (expressing the cell-associated full-length *EphA2* gene) treatment improved median and overall survival (n=8–9 mice/cohort, *p<0.026 compared with saline or C172-treated mice, log-rank test; p values were adjusted by Holm’s method).

### TIL immunophenotypic characterization

**Figure 4:**
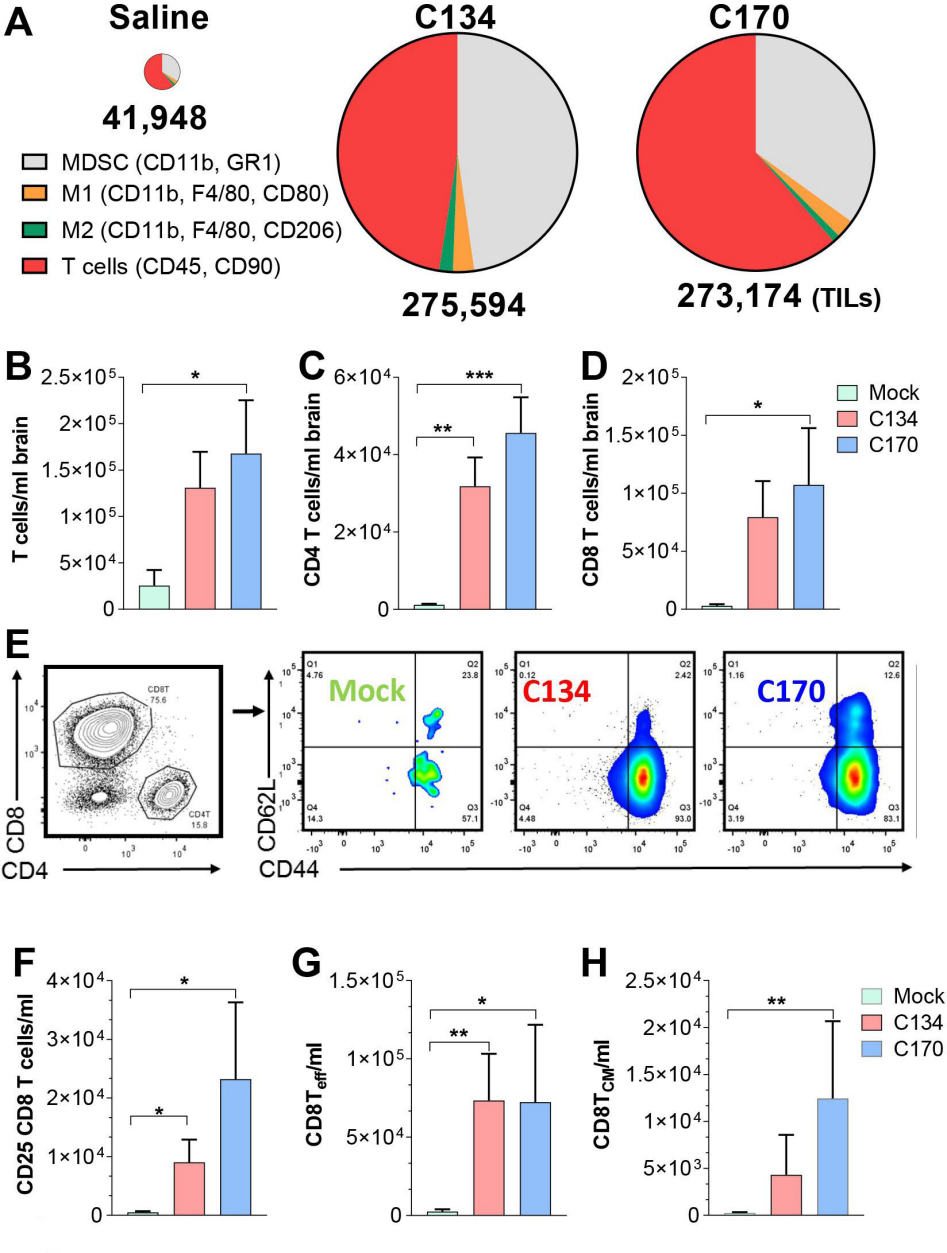
CT2A tumor infiltrate immunophenotypic analysis. (A). Representative summary of TILs and population changes 6 days post saline, C134, and C170 (C134+Epha2) treatment. Numbers below pie chart represent TILs/ml brain. (B–D). T-cell tumor infiltrate and subset analysis from saline (green), C134 (salmon), and C170 (blue)-treated mice. (E) Representative gating summary of CD8 subsets and CD62L/CD44 staining in treated mice. CD8 subset analysis of (F) CD8, CD25+, (G) CD8+, CD44+, CD62L effector-like population changes, and (H) CD8+, CD44+, CD62L+central memory-like population changes in C170-treated mice. Data were analyzed by one-way analysis of variance; p values were adjusted by Holm’s procedure. TIL: tumor-infiltrating leukocyte, MDSC: myeloid derived suppressor cells.

To identify differences in the antitumor immune activity in our different treatment cohorts, we examined the immune infiltrates from CT2A brain tumor-bearing mice. Mice were sacrificed 6 days post-treatment, and the mononuclear cell infiltrates were isolated and quantified from the saline perfused and homogenized brains as described in the Materials and methods section. The results show that OV treatment increased overall T cell and myeloid infiltrates in the brain (figure 4A). There were qualitative differences in the immune infiltrates following C134 and C170 treatment. Both viruses increased T-cell infiltration; however, C170 more often demonstrated statistically significant increases over the saline cohort (figure 4B–D). Both C134 and C170 significantly increased the CD4 T-cell infiltrates (C134 3.14×10^4^ vs saline 1.18×10^3^ (**p=0.0081), C170 4.56×10^4^ vs saline 1.18×10^3^ (**p=0.0012); figure 4C). The results also show that the majority of the T cells infiltrating after virotherapy are CD8 T cells and that C170 significantly increased the CD8 T-cell population when compared with saline-treated cohorts (C170 1.07×10^5^ vs saline 2.93×10^3^, *p=0.04; figure 4D). Further subset analysis of the CD8 infiltrates was performed and revealed additional differences between the saline and OV-treated mice (representative CD62L and CD44 subset gating is shown in figure 4E). Both oHSV-treated cohorts recruited activated T cells ([CD8+, CD25+] C134 9.05×10^3^ vs saline 5.20×10^2^, *p=0.049 and C170 2.32×10^4^ vs saline 5.20×10^2^, *p*=*0.022; figure 4F) and CD8 effector memory-like populations([CD8+, CD44+, CD62L−], C134 7.34×10^4^ vs saline 2.55×10^3^, **p=0.0074; C170 7.24×10^4^ vs saline 2.55×10^3^, *p*=*0.0284; figure 4E, G); however, only C170 significantly increased the CD8 central memory-like (CD8+, CD62L+, CD44+) population (C170 1.24×10^4^ vs saline 2.54×10^2^, **p=0.0071; figure 4E, H). Statistical analysis performed using one-way ANOVA with p values adjusted by Holm’s procedure. A summary of the gating strategy, representative flow plots, and additional comparisons (myeloid and T_REG-like_ changes) between the treatment cohorts are included in online supplemental figures 2–4.

### C170 antitumor activity and T-cell dependence

**Figure 5:**
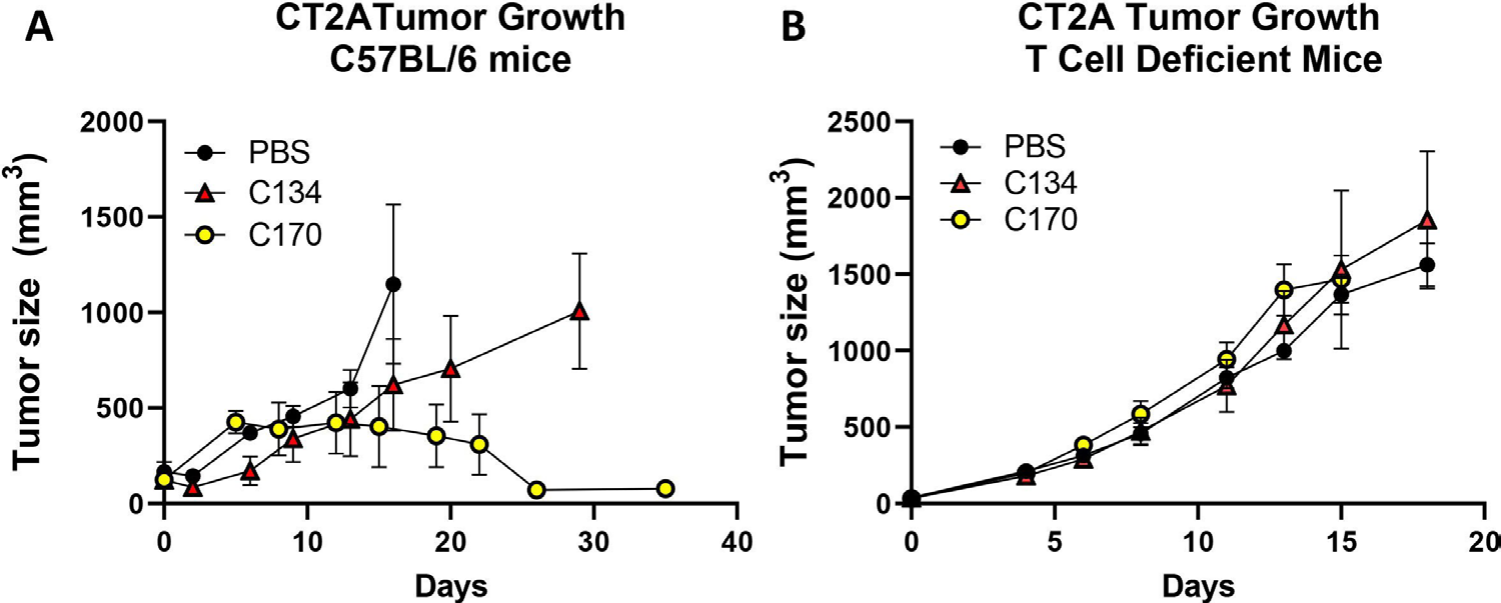
C170 treatment effect on CT2A tumor growth in immunocompetent and T cell-deficient mice. CT2A tumors were implanted in (A) immunocompetent and (B) T cell-immunodeficient mice and treated with either oHSV (C170 or C134 at 3×10^7^ PFU/50 µL) or vehicle (50 µL) and followed for tumor growth. In the immunocompetent mice, tumors initially grew in the C170 treated cohort but then regressed ~2 weeks later, whereas tumors continued to grow in the C134 and saline-treated groups. Endpoint tumor sizes show that C170 significantly reduced tumor growth compared with C134 therapy and saline controls (C170 vs saline: (****p<0.0001), C170 vs C134 (**p=0.0011: n=4). In athymic nude mice, C170 treatment had no impact on CT2A tumor growth (NS, n=6–8). NS, not significant; oHSV, oncolytic herpes simplex virus.

To assess the role of T cells in C170-mediated anti-CT2A activity, we next assessed tumor growth in immunecompetent and athymic nude mice. CT2A tumors were implanted both in immunocompetent C57BL/6 mice and athymic nude mice, and once tumors were established and achieved a size between 25 and 200 mm^3^, the athymic and immunocompetent mice were randomized into three cohorts and intratumorally injected with either vehicle or C170 or C134 at 3×10^7^ PFU in parallel studies. The results show that similar to the orthotopic studies, there was no statistically significant difference in antitumor activity between saline and C134 in the flank studies (figure 5A). In contrast, C170-treated tumors stablized and then began to decrease in size ~10 to 14 days post-treatment, and eventually the mice cleared the tumors (figure 5A). Endpoint analysis showed that C170 reduced tumor size compared with either the C134 or saline controls (saline-d16 vs C170 d35: difference=1069.2, ****p value<0.0001; C134-d29 vs C170-d35: difference=945.49, **p value=0.0011). In mice lacking T cells, C170 had no antitumor effect and behaved similarly to saline or C134-treatment (figure 5B).

## Tumor rechallenge

**Figure 6:**
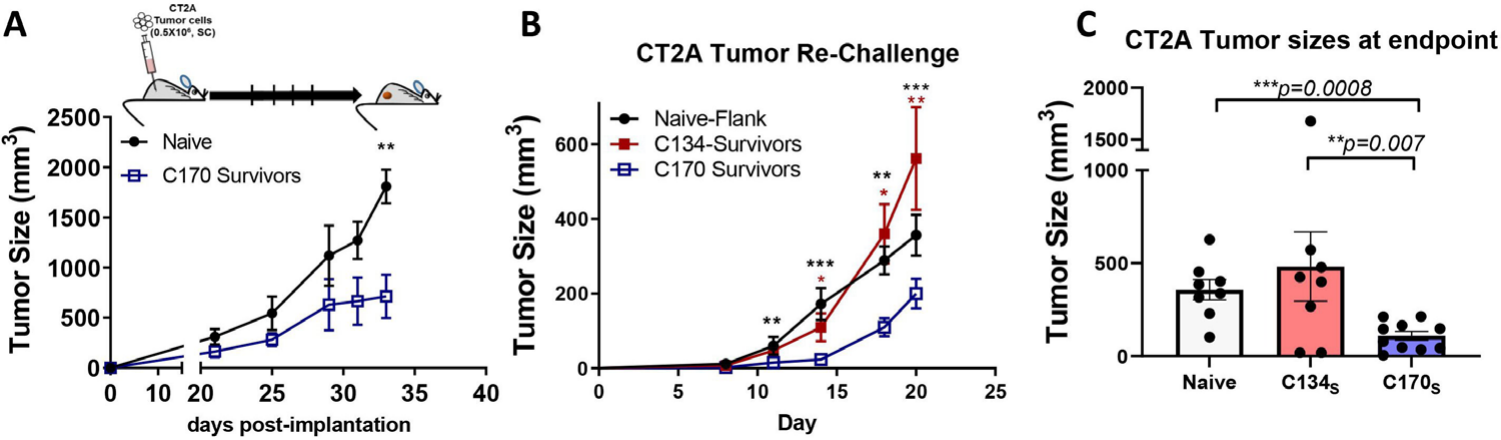
CT2A rechallenge. C134 and C170 survivors and naïve C57BL/6 mice were rechallenged with CT2A tumor cells in their flanks and tumor growth evaluated in the survivors. Results from independent experiments (A & B) show that C170 survivors suppress tumor growth better than C134 survivor mice or naïve mice. C) Analysis of the D20 timepoint from graph B. Data were first cubic root transformed to meet the normality assumption for the residuals of the model. Data were analyzed by using analysis of variance with repeated measures. P values were adjusted by Holm’s procedure.

In the orthotopic CT2A model, almost half of the C170 treated and a minority of C134 treated mice (10%–12.5%) survived in repeated experiments. Our orthotopic studies (figure 4) indicated that this was a T cell-dependent antitumor activity, and immunophenotypic analysis suggested that C170 uniquely induced a central memory-like population. To determine if there were functional differences between the C170-treated and C134-treated mice with regard to their antitumor memory, the long-term survivor population was rechallenged with CT2A tumor cells. None of these tumor-rechallenged mice underwent any virotherapy treatment. In initial studies, C170 survivor mice (n=3) from the brain tumor studies were compared with naïve mice (n=3). The results showed that C170 survivors when compared with CT2A-naïve mice signficantly restricted CT2A tumor growth rates after CT2A rechallenge (**p*=*0.00102 C170 vs naive: adjusted for multiple comparisons using the Holm-Sidak method) (figure 6A). These studies were again repeated using C170 orthotopic tumor survivors (n=5) and were compared with C134 survivor mice (n=5) pooled from all of the orthotopic tumor studies. Again the C170 survivors displayed signficantly reduced tumor growth on rechallenge when compared with either naïve or C134-treated CT2A survivors (figure 6B). The experiment ended at animal resources personnel request when a C134 long-term survivor reached endpoint. Tumor size was similar in the CT2A naïve mice and the rechallenged C134 survivors. In contrast, the C170 long-term survivors resisted tumor on rechallenge when compared with C134-treated survivors or tumor-naïve mice based on tumor growth and tumor size distributions at experimental endpoint (C170 survivors vs naïve (*p*=*0.0225) and C170 survivors vs C134 survivors (*p*=*0.0450), Kruskal Wallis test with p values adjusted using Dunn’s test for multiple comparisons) (figure 6B, C).

### Evaluation in an alternative *EphA2* (+) tumor model

**Figure 7:**
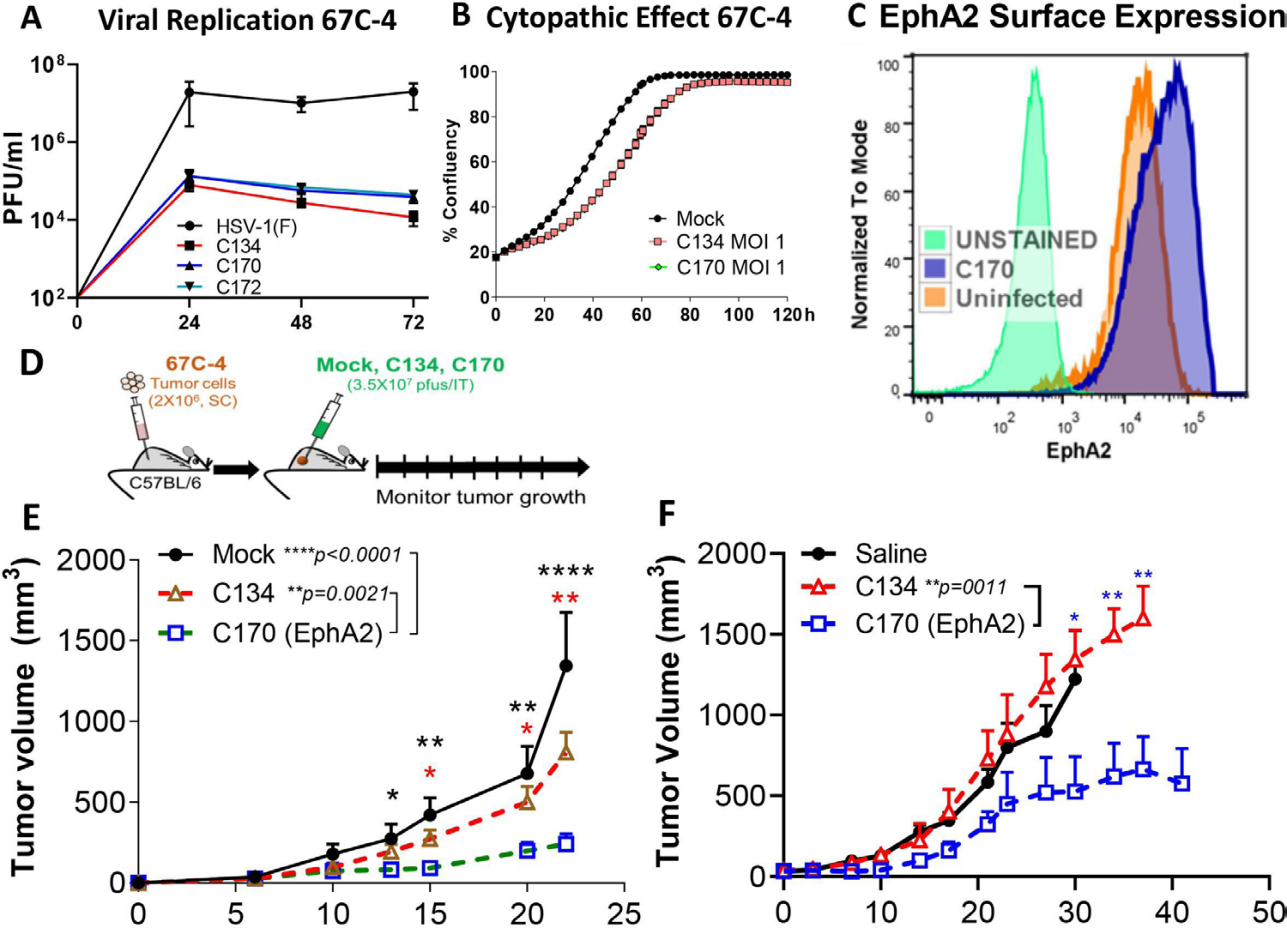
Summary of 67C-4-oHSV antitumor response studies. C170 was evaluated in vitro and showed equivalent (A) replication and (B) cytopathic activity as the parent virus C134. (C) 67 C-4 expresses EphA2 on the cell surface in mock and C170 infected cells. (D) Schematic summary of experimental approach. (E&F) Independent studies show C170 virotherapy treatment of established 67 C-4 flank tumors shows that C170 significantly suppresses tumor growth when compared with saline or C134-treated cohorts. Data were analyzed by using analysis of variance with repeated measures. P values were adjusted by Holm’s procedure.

Taken together, the brain tumor results indicated that the antigen-expressing virus induced a T cell-dependent antitumor population that persisted and reduced tumor growth on rechallenge. To determine if this was unique to this one tumor model, we next examined C170 activity in an *EphA2* (+) syngeneic MPNST model. Our previous studies showed that the immunocompetent 67 C-4 tumors resisted oHSV replication and oHSV antitumor activity.^40^ Similar to the CT2A studies, we first examined oHSV replication and *EphA2* expression’s effect on cell proliferation in 67 C-4 cell culture. Consistent with the CT2A studies, C170 had no replication or cytopathic advantage over the parent C134 virus (figure 7A, B). Similar to the earlier CT2A studies, infected and uninfected 67 C-4 tumor cells express abundant EphA2 on their surface (figure 7C). Next, to determine how oHSV *EphA2* expression improved antitumor response, tumor growth studies were performed. Similar to our previous studies, we implanted 2×10^6^ 67 C-4 cells in the flanks of C57BL/6 mice.^13^ Once the tumors were of sufficient size (25–200 mm^3^), mice were randomized into three treatment cohorts and treated with saline or a single dose (3×10^7^ PFU) of C134 or C170 as summarized in figure 7D. These studies were repeated for biological reproducibility and are summarized in figure 7E, F. In brief, C134 had no antitumor effect in this resistant tumor model, consistent with our previous studies.^40^ In contrast, treatment with the C134-based *EphA2* expressing virus (C170) signficanctly reduced tumor growth in comparison to C134 treatment (C170 (n=7) vs C134 (n=8), p=0.0021; C170 (n=7) vs saline (n=6), p<0.0001; figure 7E; C170 (n=7) vs C134 (n=10), p=0.0011; adjusted p value by pairwise analysis (Tukey-Kramer); figure 7F).

### EphA2-specific response

**Figure 8:**
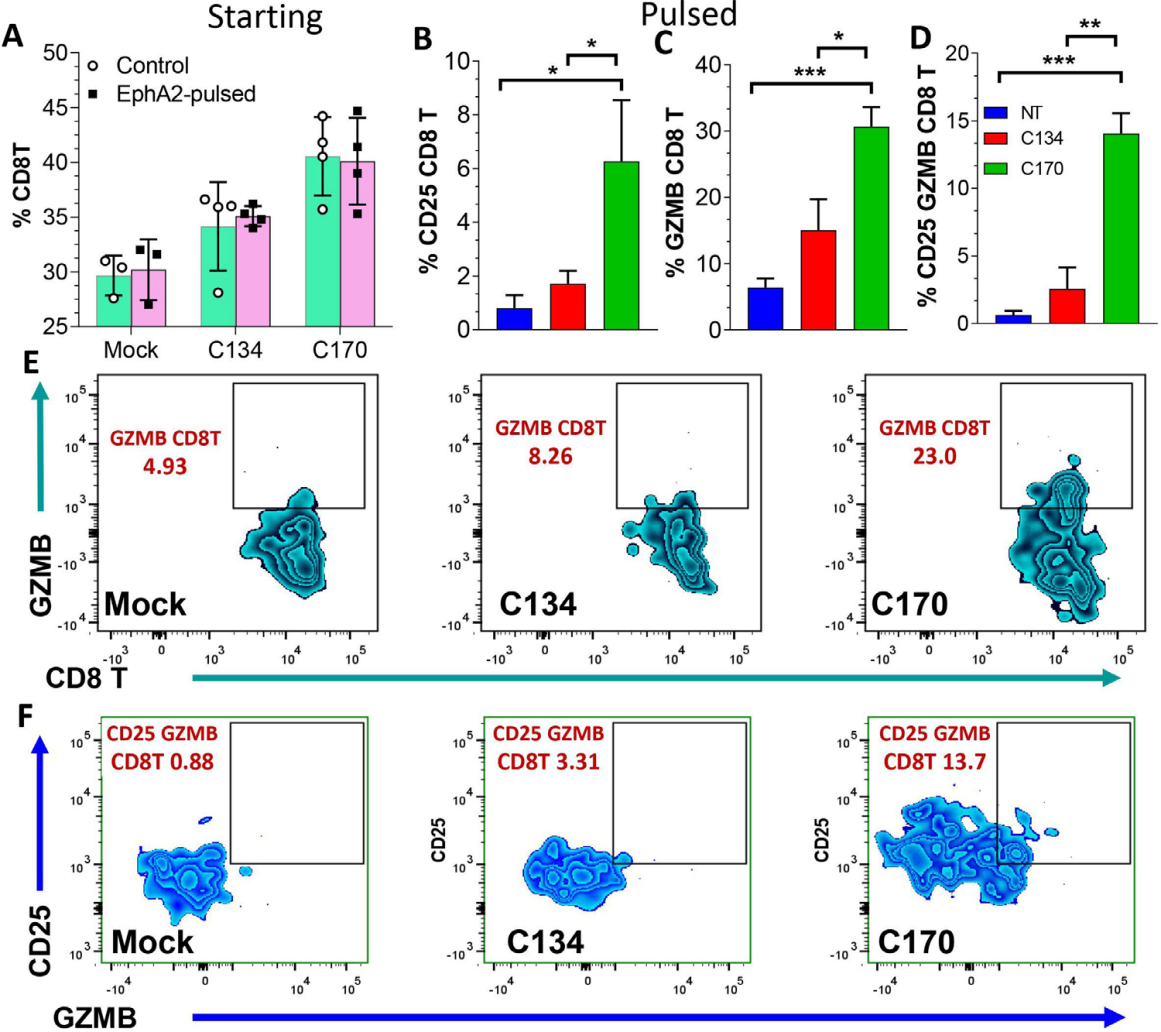
T-cell function studies from saline and oHSV treated mice shows that C170 treatment induces an antigen-specific T-cell response in the periphery of long-term survivors. Splenocytes from saline (blue column) or oHSV-treated mice (red column, C134; green column, C170) were analyzed. (A) At the start, there was no difference in the populations that used peptide pulsing; however, after pulsing with 10 µm EphA2 or 10µM OVA peptide (negative control), C170-treated mice significantly increase their activated (B) CD25(+), (C) GZMB(+), and (D) CD25+, GZMB+ dual staining CD8+ populations indicative of an EphA2-specific population response. (E) Representative flow plot showingCD8(+) GZMB(+) population and gating, and the (F) GZMB CD25 dual-positive populations. oHSV, oncolytic herpes simplex virus; OVA, ovalbumin.

The rechallenge studies from CT2A mice (figure 6) suggested that C170 and C134 therapy differ in their functional response and that C170 induced a long-lasting T-cell population that was tumor antigen-specific. To further investigate the T-cell functional response and to determine if this immune activity was directed against the virus-expressed antigen, we took splenocytes from the mock and oHSV-treated 67 C-4 mice at the conclusion of the tumor growth studies and analyzed their peripheral T-cell response by peptide pulsing. The mock, C134-treated and C170-treated survivor splenocytes were exposed to no protein and to 10 µm *EphA2* peptide or *ovalbumin* (*OVA*) peptide negative control for 6 hours and analyzed by flow cytometry for intracellular *granzyme B* (*GZMB*) staining and CD25 upregulation. The results show that at the start of pulsing, there was no difference in T-cell activity in the two pulsed populations (figure 8A); however, after pulsing with 10 µm *EphA2* or 10 µM OVA peptide (negative control), splenocytes from the C170-treated mice significantly increased their activated CD25 (+), *GZMB* (+), and CD25, GZMB dual staining CD8+ populations (figure 8B–D) indicative of an *EphA2*-specific population response. Representative flow cytometry plots showing the CD8 (+), *GZMB* (+) (figure 8E), and GZMB CD25 dual-positive CD8 populations (figure 8F) were provided. In contrast, neither saline nor C134-treatment induced this *EphA2* reactive T-cell population. There was no difference in the treatment cohorts when exposed to OVA control peptide, further suggesting that this T-cell response was specific to the viral expressed *EphA2* and not the result of generalized T-cell activity after C170 treatment (online supplemental figure 5). A summary of the gating strategy, representative flow plots, and additional comparisons following peptide pulsing studies (eg, GZMB/CD25, GZMB/IFN-γ, and TNF-α/IFN-γ dual-positive staining) of the antitumor and antiviral responses is included in online supplemental figures 6–9.

## Discussion

These studies were initiated to identify if further viral modifications could improve OV antitumor activity specifically for OV-resistant low-mutational load tumors. We postulated that by engineering the virus to encoding a tumor-associated shared antigen, we could bypass viral host *EphA2* transcriptional downregulation and improve immune recognition of that shared antigen during tumor infection. For these studies, we chose a next-generation OV as our platform because it had improved protein translation and stimulated an IFN, chemokine, and cytokine response.^38 44^ We anticipated that this would provide an ideal multimodal therapeutic platform. Our results show that in two different C57BL/6 immune-competent tumor models that possess high basal *EphA2* expression, OV treatment alone did not elicit an immune-mediated antitumor response. However, an oHSV engineered to express the C57BL/6 EphA2 antigen improved survival and memory response suppressing tumor growth on rechallenge in survivors or induced CD8 cells with improved antigen response. Our results also show that oHSV-based *Epha2* expression recruits immune cells to the tumor similar to the parent virus but that the antigen-expressing virus induces antigen-specific immune cells (CD8 T cells) that target the expressed tumor antigens. We tested whether this was unique to this brain tumor model (CT2A brain tumors) or whether a similar activity occurred in an *EphA2* (+) syngeneic peripheral sarcoma tumor model. Our results showed a similar improvement suggesting that this was not limited to a single Central Nervous System (CNS) tumor model. Like the brain tumor model, antitumor-specific T cells were examined in the periphery and again showed a functional response against tumor antigens that the original parent oHSV was unable to elicit in these two oHSV-resistant tumor models. Based on these findings, we propose that oHSVs modified to express shared tumor antigens can elicit an antitumor antigen-specific immune response and improve durability of antitumor activity.

Not all cancers provide good immune targets.^25^ Multiple mechanisms can contribute to making these tumors ‘cold’ or resistant to immunotherapy. These include lower mutational rates, reduced lymphocyte cell infiltrates, low MHC expression and elevated numbers of immunosuppressive cellular infiltrates.^45–47^ Virotherapy enhances immune cell infiltration into the tumor and MHC upregulation and can change myeloid polarization, all of which can turn a ’cold’ tumor to ‘hot’.^25 48^ However, if these tumors have low mutational rates and no neoantigens, the immune cells may not generate ongoing antitumor activity after viral replication ceases. The immune cells produce memory populations against the viral antigens and are unlikely to recognize tumor antigens. Our new approach can improve immune activity against specific TAA self-antigens and provides a new strategy for tumors with low mutation rates. This is especially important for pediatric cancers because they are often driven by transcriptional changes (fusion mutations or ectopic oncogene expression) and have lower mutational loads than adult cancers.^49–51^ They are also less responsive to many immunotherapies.^25^ Our approach provides a new immunotherapeutic strategy to target tumors with low neoantigen loads that were previously resistant to immunotherapeutic approaches. This is an important mechanistic discovery for treating pediatric cancer because it indicates that an oncolytic virus can break immune tolerance and functionally vaccinate the patient against TAAs concurrent with OV.

In our two tumor models, C170 treatment suppressed tumor growth and induced memory and effector activity as evidenced by the functional tumor suppression on rechallenge and functional effector activity (CD25, IFNγ and GZMB (Granzyme B) Intracranial (IC) staining) in response to *EphA2* peptide pulsing. C170 therapy increased the T cell and CD8 effector infiltrates. While both OVs induced immune effectors, the antigen-expressing virus, C170, more effectively altered this balance and improved TAA recognition. Taken together, our phenotypic and pulsing studies suggest that, while similar immune cells are recruited to the OV-treated tumors, the antigen-expressing OV changes their function and it is this functional characterization that is more important.

There are several important scientific questions that are left unanswered by these studies. The first involves further elucidating the immune components required for a successful antigen-specific response after virus-based antigen expression. Our tumor rechallenge and peptide pulsing studies suggested that C170 uniquely elicited a tumor-associated antigenic response that protected the mice. This combined with the initial antitumor survival differences between the C172-treated and C170-treated cohorts suggested that the secreted extracellular portion of the Epha2 provided less antigenic motifs than the full length protein (C170) in C57BL/6 mice. The current data suggest that this involves an MHC I directed CD8 effector response, but these data do not exclude the possibility that virus-based antigen expression involves an alternative antigen presentation pathway that is more effective for long-term immunity. Both of our tested tumors had abundant MHC I surface expression (online supplemental figure 10); virus-infected cells frequently present antigens for immune recognition through MHC I. To further investigate this possibility, we performed C57BL/6-based *MHC I* antigen modeling (online supplemental figure 11). The antigenic domains predicted to be expressed as class I peptides for CD8 functional response lie within the intracellular portion of the *EphA2* protein predominantly, and this was consistent with our C170 and C172 brain tumor response results. Only the C170 OV was able to produce an immune-mediated antitumor response and memory protection. In contrast, C172 (expressing the extracellular *EphA2* domain) did not generate an antitumor response or improve survival. One potential explanation is that the extracellular portion of the Epha2 provided less antigenic motifs than the full-length protein in B6 mice based on the MHCI predictive modeling. Future studies will further examine how viral antigen expression induces this improved immune recognition of the shared antigen and whether the antigenic response is limited to the virus-expressed antigen or whether additional antitumor antigen recognition occurs (epitope spread).

Another question raised by our results is whether this in vivo vaccination strategy applies to other shared tumor antigens or whether *EphA2* is unique in its ability to stimulate this immune recognition after OV expression. If OV expression of other TAAs improves immune recognition, then this could lead to a new OV class encoding polyvalent tumor antigens. This would provide several translational benefits as a therapy. It would be less expensive to produce current good manufacturing practice virus that could be used for multiple tumor types and, in the case of tumors that express multiple shared antigens, could produce a polyvalent response and reduce the chances of antigenic escape. This approach, however, could also increase autoimmune complication risks.

A third area of interest is whether targeting shared antigens induces persistent autoimmunity. Again, further elucidation of the cytokine and immune cell mechanisms important for breaking immune tolerance, along with the choice of antigen will be important. Evaluating autoimmunity risk is essential for any immunotherapeutic strategy, and oHSV-based TAA expression requires further assessment before declaring this as a clinically tenable therapeutic approach. Furthermore, there may be differences between oncolytic viruses with regard to how they induce autoimmunity. In the case of HSV autoimmune disease, peripheral tolerance usually re-establishes following lytic infection but is then subverted with subsequent viral reactivation events. This may not be the case for other viruses that induce autoimmune responses through molecular mimicry (eg, measles and autoimmune encephalitis).^52 53^ Another area of interest is how to extend this in vivo vaccination approach. We anticipate that pairing the TAA expressing oHSV with other immunotherapeutic approaches such as targeting coinhibitory proteins (eg, *PD1* or *PDL1*) or combined virus-based T cell-activating cytokine expression (eg, *IL12*, *IL18*, *IFN-γ*) will enhance and extend this antigen-specific antitumor immune response.

Our current results suggest that oHSV-based expression of shared tumor antigens provides an immunotherapeutic strategy by which we can target tumors resistant to viroimmunotherapy. This is an important mechanistic discovery for treating pediatric cancer or low-mutational load adult cancers because it indicates that using our oncolytic viral platform, we can break immune tolerance and functionally vaccinate the patient against TAAs during OV. Future studies will determine if this response can be improved through repeated dosing (prime boost) against the antigen and can increase epitope spread.

## Data Availability

Data sharing not applicable as no datasets generated and/or analyzed for this study. Data are available upon reasonable request.

## References

[1] Anita Koshy G G, Avigan D. Neoantigen-based vaccines as a promising strategy in cancer immunotherapeutics. ImmunoMedicine 2021;00:e1021.

[2] Lim AR, Rathmell WK, Rathmell JC. The tumor microenvironment as a metabolic barrier to effector T cells and immunotherapy. Elife 2020;9:e55185. 10.7554/eLife.55185 32367803 PMC7200151

[3] Hassan A, Pestana RC, Parkes A. Systemic options for malignant peripheral nerve sheath tumors. Curr Treat Options Oncol 2021;22:33. 10.1007/s11864-021-00830-7 33641042

[4] Foreman PM, Friedman GK, Cassady KA, et al. Oncolytic virotherapy for the treatment of malignant glioma. Neurotherapeutics 2017;14:333–44. 10.1007/s13311-017-0516-0 28265902 PMC5398989

[5] Lin L, Cai J, Jiang C. Recent advances in targeted therapy for glioma. Curr Med Chem 2017;24:1365–81. 10.2174/0929867323666161223150242 28019637

[6] Maire CL, Mohme M, Bockmayr M, et al. Glioma escape signature and clonal development under immune pressure. J Clin Invest 2020;130:5257–71. 10.1172/JCI138760 32603315 PMC7524465

[7] Reilly KM, Kim A, Blakely J, et al. Neurofibromatosis type 1-associated MPNST state of the science: Outlining a research agenda for the future. J Natl Cancer Inst 2017;109:1. 10.1093/jnci/djx124 PMC6057517 29117388

[8] Martin E, Lamba N, Flucke UE, et al. Non-cytotoxic systemic treatment in malignant peripheral nerve sheath tumors (MPNST): a systematic review from bench to bedside. Crit Rev Oncol Hematol 2019;138:223–32. 10.1016/j.critrevonc.2019.04.007 31092379

[9] Cheever MA, Allison JP, Ferris AS, et al. The prioritization of cancer antigens: a national cancer institute pilot project for the acceleration of translational research. Clin Cancer Res 2009;15:5323–37. 10.1158/1078-0432.CCR-09-0737 19723653 PMC5779623

[10] Liu XS, Mardis ER. Applications of Immunogenomics to cancer. Cell 2017;168:600–12. 10.1016/j.cell.2017.01.014 28187283 PMC5972371

[11] Gros A, Parkhurst MR, Tran E, et al. Prospective identification of neoantigen-specific lymphocytes in the peripheral blood of melanoma patients. Nat Med 2016;22:433–8. 10.1038/nm.4051 26901407 PMC7446107

[12] Endo Y, Lyon S, Shen Y, et al. Cell proliferation and invasion are regulated differently by EGFR and MRP1 in T-DM1-resistant breast cancer cells. Sci Rep 2019;9:16383. 10.1038/s41598-019-52797-z 31704984 PMC6842003

[13] Shi X, Wang B. Caught in the "Akt": Cross-talk between EphA2 and EGFR through the Akt-PIKfyve axis maintains cellular sensitivity to EGF. Sci Signal 2018;11 10.1126/scisignal.aau1207 30065027 PMC6311088

[14] Baharuddin WNA, Yusoff AAM, Abdullah JM, et al. Roles of EphA2 receptor in angiogenesis signaling pathway of glioblastoma multiforme. Malays J Med Sci 2018;25:22–7. 10.21315/mjms2018.25.6.3 30914876 PMC6422564

[15] Zhou Y, Sakurai H. Emerging and diverse functions of the EphA2 noncanonical pathway in cancer progression. Biol Pharm Bull 2017;40:1616–24. 10.1248/bpb.b17-00446 28966234

[16] Kisielow P. How does the immune system learn to distinguish between good and evil? The first definitive studies of T cell central tolerance and positive selection. Immunogenetics 2019;71:513–8. 10.1007/s00251-019-01127-8 31418051 PMC6790186

[17] Darling TK, Lamb TJ. Emerging roles for Eph receptors and ephrin ligands in immunity. Front Immunol 2019;10:1473. 10.3389/fimmu.2019.01473 31333644 PMC6620610

[18] Biao-xue R, Xi-guang C, Shuan-ying Y, et al. EphA2-dependent molecular targeting therapy for malignant tumors. Curr Cancer Drug Targets 2011;11:1082–97. 10.2174/156800911798073050 21933105

[19] Park JE, Son AI, Zhou R. Roles of EphA2 in development and disease. Genes 2013;4:334–57. 10.3390/genes4030334 24705208 PMC3924825

[20] Xiao T, Xiao Y, Wang W, et al. Targeting EphA2 in cancer. J Hematol Oncol 2020;13:114. 10.1186/s13045-020-00944-9 32811512 PMC7433191

[21] Wykosky J, Debinski W. The EphA2 receptor and EphrinA1 ligand in solid tumors: function and therapeutic targeting. Mol Cancer Res 2008;6:1795–806. 10.1158/1541-7786.MCR-08-0244 19074825 PMC3690928

[22] Cioce M, Fazio VM. Epha2 and EGFR: friends in life, partners in crime. can EphA2 be a predictive biomarker of response to anti-EGFR agents? Cancers 2021;13:700. 10.3390/cancers13040700 33572284 PMC7915460

[23] Hong HN, Won YJ, Shim JH, et al. Cancer-associated fibroblasts promote gastric tumorigenesis through EphA2 activation in a ligand-independent manner. J Cancer Res Clin Oncol 2018;144:1649–63. 10.1007/s00432-018-2683-8 29948146 PMC11813431

[24] Miao H, Li D-Q, Mukherjee A, et al. Epha2 mediates ligand-dependent inhibition and ligand-independent promotion of cell migration and invasion via a reciprocal regulatory loop with Akt. Cancer Cell 2009;16:9–20. 10.1016/j.ccr.2009.04.009 19573808 PMC2860958

[25] Hutzen B, Ghonime M, Lee J, et al. Immunotherapeutic challenges for pediatric cancers. Mol Ther Oncolytics 2019;15:38–48. 10.1016/j.omto.2019.08.005 31650024 PMC6804520

[26] Getts DR, Chastain EML, Terry RL, et al. Virus infection, antiviral immunity, and autoimmunity. Immunol Rev 2013;255:197–209. 10.1111/imr.12091 23947356 PMC3971377

[27] Nauclér CS, Larsson S, Möller E. A novel mechanism for virus-induced autoimmunity in humans. Immunol Rev 1996;152:175–92. 10.1111/j.1600-065X.1996.tb00916.x 8930673

[28] Wickham S, Carr DJJ. Molecular mimicry versus bystander activation: herpetic stromal keratitis. Autoimmunity 2004;37:393–7. 10.1080/08916930410001713106 15621563

[29] Banerjee K, Biswas PS, Kumaraguru U, et al. Protective and pathological roles of virus-specific and bystander CD8+ T cells in herpetic stromal keratitis. J Immunol 2004;173:7575–83. 10.4049/jimmunol.173.12.7575 15585885

[30] Armangue T, Spatola M, Vlagea A, et al. Frequency, symptoms, risk factors, and outcomes of autoimmune encephalitis after herpes simplex encephalitis: a prospective observational study and retrospective analysis. Lancet Neurol 2018;17:760–72. 10.1016/S1474-4422(18)30244-8 30049614 PMC6128696

[31] Da Ros M, De Gregorio V, Iorio AL, et al. Glioblastoma chemoresistance: the double play by microenvironment and blood-brain barrier. Int J Mol Sci 2018;19 10.3390/ijms19102879 30248992 PMC6213072

[32] Truong NR, Smith JB, Sandgren KJ, et al. Mechanisms of immune control of mucosal HSV infection: a guide to rational vaccine design. Front Immunol 2019;10:373. 10.3389/fimmu.2019.00373 30894859 PMC6414784

[33] Shu M, Taddeo B, Zhang W, et al. Selective degradation of mRNAs by the HSV host shutoff RNase is regulated by the UL47 tegument protein. Proc Natl Acad Sci U S A 2013;110:E1669–75. 10.1073/pnas.1305475110 23589852 PMC3645526

[34] Saffran HA, Read GS, Smiley JR. Evidence for translational regulation by the herpes simplex virus virion host shutoff protein. J Virol 2010;84:6041–9. 10.1128/JVI.01819-09 20357089 PMC2876651

[35] Früh K, Ahn K, Djaballah H, et al. A viral inhibitor of peptide transporters for antigen presentation. Nature 1995;375:415–8. 10.1038/375415a0 7760936

[36] Smiley JR. Herpes simplex virus virion host shutoff protein: immune evasion mediated by a viral RNase? J Virol 2004;78:1063–8. 10.1128/JVI.78.3.1063-1068.2004 14722261 PMC321390

[37] Shah AC, Parker JN, Gillespie GY, et al. Enhanced antiglioma activity of chimeric HCMV/HSV-1 oncolytic viruses. Gene Ther 2007;14:1045–54. 10.1038/sj.gt.3302942 17429445

[38] Cassady KA. Human cytomegalovirus TRS1 and IRS1 gene products block the double-stranded-RNA-activated host protein shutoff response induced by herpes simplex virus type 1 infection. J Virol 2005;79:8707–15. 10.1128/JVI.79.14.8707-8715.2005 15994764 PMC1168740

[39] Ghonime MG, Jackson J, Shah A, et al. Chimeric HCMV/HSV-_1_ and Δγ_1_34.5 oncolytic herpes simplex virus elicit immune mediated antigliomal effect and antitumor memory. Transl Oncol 2018;11:86–93. 10.1016/j.tranon.2017.10.005 29216507 PMC6002352

[40] Ghonime MG, Cassady KA. Combination therapy using ruxolitinib and oncolytic HSV renders resistant MPNSTs susceptible to virotherapy. Cancer Immunol Res 2018;6:1499–510. 10.1158/2326-6066.CIR-18-0014 30352799

[41] Hatano M, Kuwashima N, Tatsumi T, et al. Vaccination with EphA2-derived T cell-epitopes promotes immunity against both EphA2-expressing and EphA2-negative tumors. J Transl Med 2004;2:40. 10.1186/1479-5876-2-40 15563374 PMC535538

[42] Mueller SN, Heath W, McLain JD, et al. Characterization of two TCR transgenic mouse lines specific for herpes simplex virus. Immunol Cell Biol 2002;80:156–63. 10.1046/j.1440-1711.2002.01071.x 11940116

[43] Hsu JC. Multiple comparison: theory and methods. London: Chapman & Hall, 1996.

[44] Cassady KA, Saunders U, Shimamura M. Δγ₁134.5 herpes simplex viruses encoding human cytomegalovirus IRS1 or TRS1 induce interferon regulatory factor 3 phosphorylation and an interferon-stimulated gene response. J Virol 2012;86:610–4. 10.1128/JVI.05099-11 22072777 PMC3255867

[45] Laccetti AL, Subudhi SK. Immunotherapy for metastatic prostate cancer: immuno-cold or the tip of the iceberg? Curr Opin Urol 2017;27:566–71. 10.1097/MOU.0000000000000433 28825923 PMC6351068

[46] Ghosh D, Nandi S, Bhattacharjee S. Combination therapy to checkmate glioblastoma: clinical challenges and advances. Clin Transl Med 2018;7:33. 10.1186/s40169-018-0211-8 30327965 PMC6191404

[47] Collins JM, Redman JM, Gulley JL. Combining vaccines and immune checkpoint inhibitors to prime, expand, and facilitate effective tumor immunotherapy. Expert Rev Vaccines 2018;17:697–705. 10.1080/14760584.2018.1506332 30058393 PMC8262093

[48] Sprague L, Braidwood L, Conner J, et al. Please stand by: how oncolytic viruses impact bystander cells. Future Virol 2018;13:671–80. 10.2217/fvl-2018-0068 30416535 PMC6219440

[49] Campbell BB, Light N, Fabrizio D, et al. Comprehensive analysis of hypermutation in human cancer. Cell 2017;171:1042–56. 10.1016/j.cell.2017.09.048 29056344 PMC5849393

[50] Amayiri N, Al-Hussaini M, Swaidan M, et al. Synchronous glioblastoma and medulloblastoma in a child with mismatch repair mutation. Child’s Nervous System 2016;32:553–7. 10.1007/s00381-015-2883-3 26293676

[51] Zhang J, Walsh MF, Wu G, et al. Germline mutations in predisposition genes in pediatric cancer. N Engl J Med 2015;373:2336–46. 10.1056/NEJMoa1508054 26580448 PMC4734119

[52] Griffin DE. Measles virus and the nervous system. Handb Clin Neurol 2014;123:577–90. 10.1016/B978-0-444-53488-0.00027-4 25015505

[53] Buchanan R, Bonthius DJ. Measles virus and associated central nervous system sequelae. Semin Pediatr Neurol 2012;19:107–14. 10.1016/j.spen.2012.02.003 22889539

