# Supplementary material for: Eliciting an immune-mediated antitumor response through oncolytic herpes simplex virus-based shared antigen expression in tumors resistant to viroimmunotherapy
Source: J Immunother Cancer. 2021 Oct 1;9(10):e002939. doi: 10.1136/jitc-2021-002939 (PMC8488720; doi:10.1136/jitc-2021-002939)
Supplement: Supplementary data [file jitc-2021-002939supp002.pdf]

Figures are included in the text and reiterated below

Supplemental Figure 1: **Histogram showing surface EphA2 expression at baseline in CT2A cells and following C134 (Parent) and C170 (C134+EphA2) infection (MOI 3).** CT2A cells infected with EpHA2 expressing C170 virus (MOI=3) stained with EphA2-PE antibody showed the highest EpHA2 expression by flow cytometry compared to PBS, C134 or the Isotype controls.

Supplemental Figure 2: Summary of Gating Strategy/Workflow for CT2A orthotopic immune phenotypic analysis used in Figure 4 and Supplemental Figures 3 & 4.

Supplemental Figure 3: Representative flow plots for MDSCs, M1, M2 population changes. Both the OV therapies increase the GR1/CD11b + Granulocyte/MDSCs populations. There was no difference between treatment groups with respect to M1 populations but C170 decreases the M2 (F4/80+, CD206+) populations.

Supplemental Figure 4: T_{REG}-like cell infiltrates in Saline and oHSV treated cohorts. oHSV treatment (C134 and C170) reduced $CD4^{+}CD25^{+}$ T_{REG}-like cell proportions (%) in CT2A orthotopic tumors.

Supplemental Figure 5: Negative control OVA peptide pulsing. No significant differences were observed amongst the OVA peptide pulsed untreated or oncolytic virus treated (C134, C170) cohorts in terms CD25 GZMB CD8T (%) populations.

Supplemental Figure 6: 67-C4 Peptide-pulsed CD8 analysis summary of gating strategy and workflow

Supplemental Figure 7: CD25, GZMB dual-positive CD8T after peptide stimulation. Peptide pulsing

results show that EphA2 peptide treatment significantly increases CD25/GZMB dual + CD8 T-cell populations in the C170 treated cohort indicating improved EphA2 recognition. C134 treatment does not significantly increase the response over saline treated controls.

Supplemental Figure 8: IFNɣ /GZMB dual positive CD8T after peptide stimulation. Peptide pulsing results for Mock, C134 and C170 treated mice show that C134 treated mice have higher baseline (IFNγ/GZMB) activity compared to saline treated mice but that neither gB or EphA2 generate a specific increase in this population. In contrast, C170-treated mice generate significantly higher CD8 T-cell response (IFNγ/GZMB) after EphA2 pulsing suggesting improved EphA2 recognition.

Supplemental Figure 9: IFNɣ /TNFα positive CD8T. Peptide pulsing results for Mock, C134 and C170 treated mice show that the oncolytic virus treated cohorts (C134 and C170) generate significant CD8, TNFα (+) subpopulation changes from IFNg (+) T cell population following gB peptide (middle panel) stimulation.

Supplemental Figure 10: MHC I expression on CT2A or 67C-4 cells relative to Isotype. CT2A or 67C-4 cells were stained with MHC I-FITC or isotype antibody. Both the cell lines showed high MHCI as measured by flow cytometry.

Supplemental Figure 11: *Ephrin A2* MHCI antigenic peptide affinity modeling showing that the predicted C57BL/6 MHC I-associated antigenic domains for the C57BL/6 EphA2 protein predominate in the cytoplasmic region.
