# Supplementary material for: Eliciting an immune-mediated antitumor response through oncolytic herpes simplex virus-based shared antigen expression in tumors resistant to viroimmunotherapy
Source: J Immunother Cancer. 2021 Oct 1;9(10):e002939. doi: 10.1136/jitc-2021-002939 (PMC8488720; doi:10.1136/jitc-2021-002939)
Supplement: Supplementary data [file jitc-2021-002939supp001.pdf]

BMJ Publishing Group Limited (BMJ) disclaims all liability and responsibility arising from any reliance placed on this supplemental material which has been supplied by the author(s)

Supplemental Figures

BMJ Publishing Group Limited (BMJ) disclaims all liability and responsibility arising from any reliance placed on this supplemental material which has been supplied by the author(s)

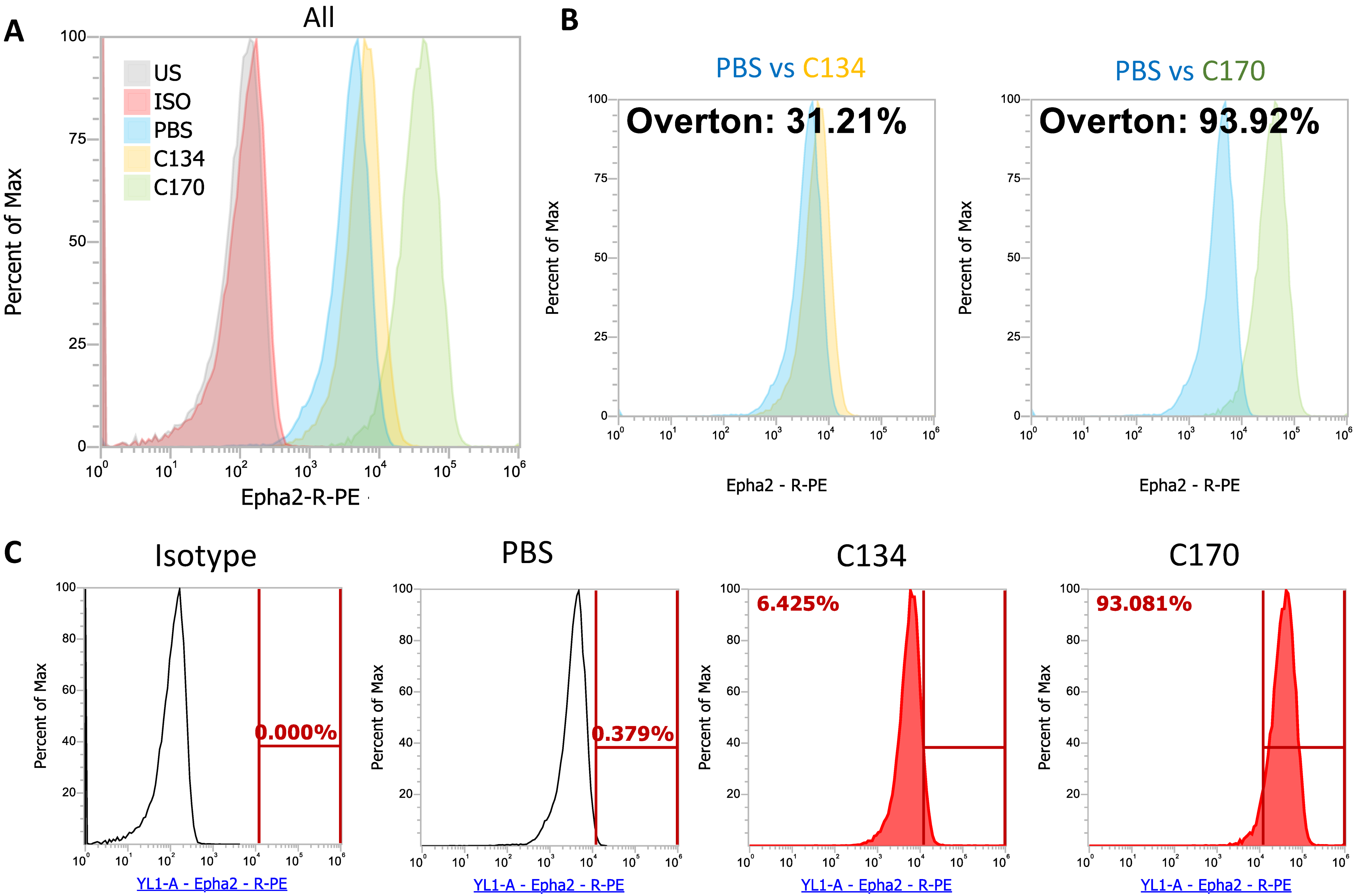

Supplemental Figure 1

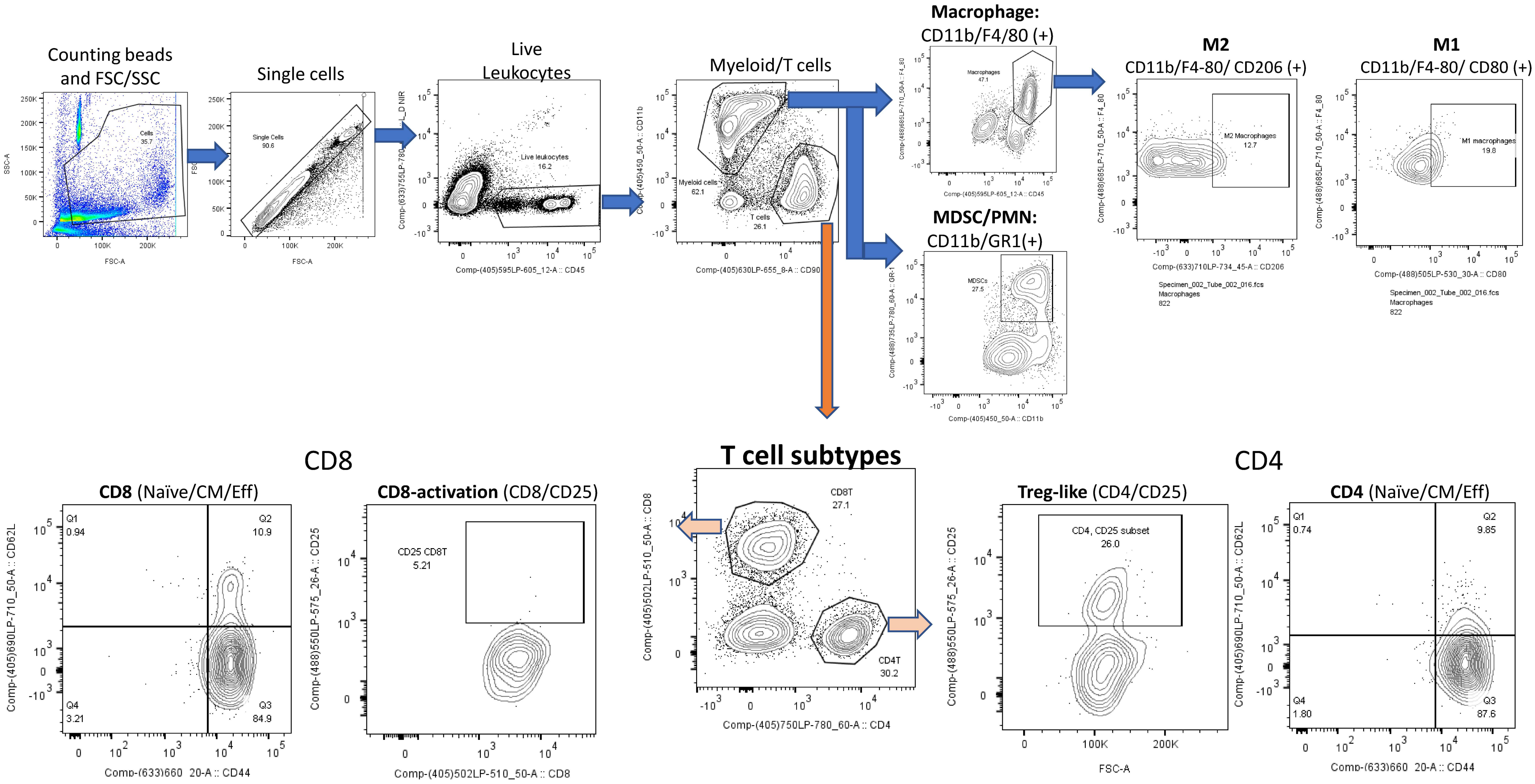

Supplemental Figure 2

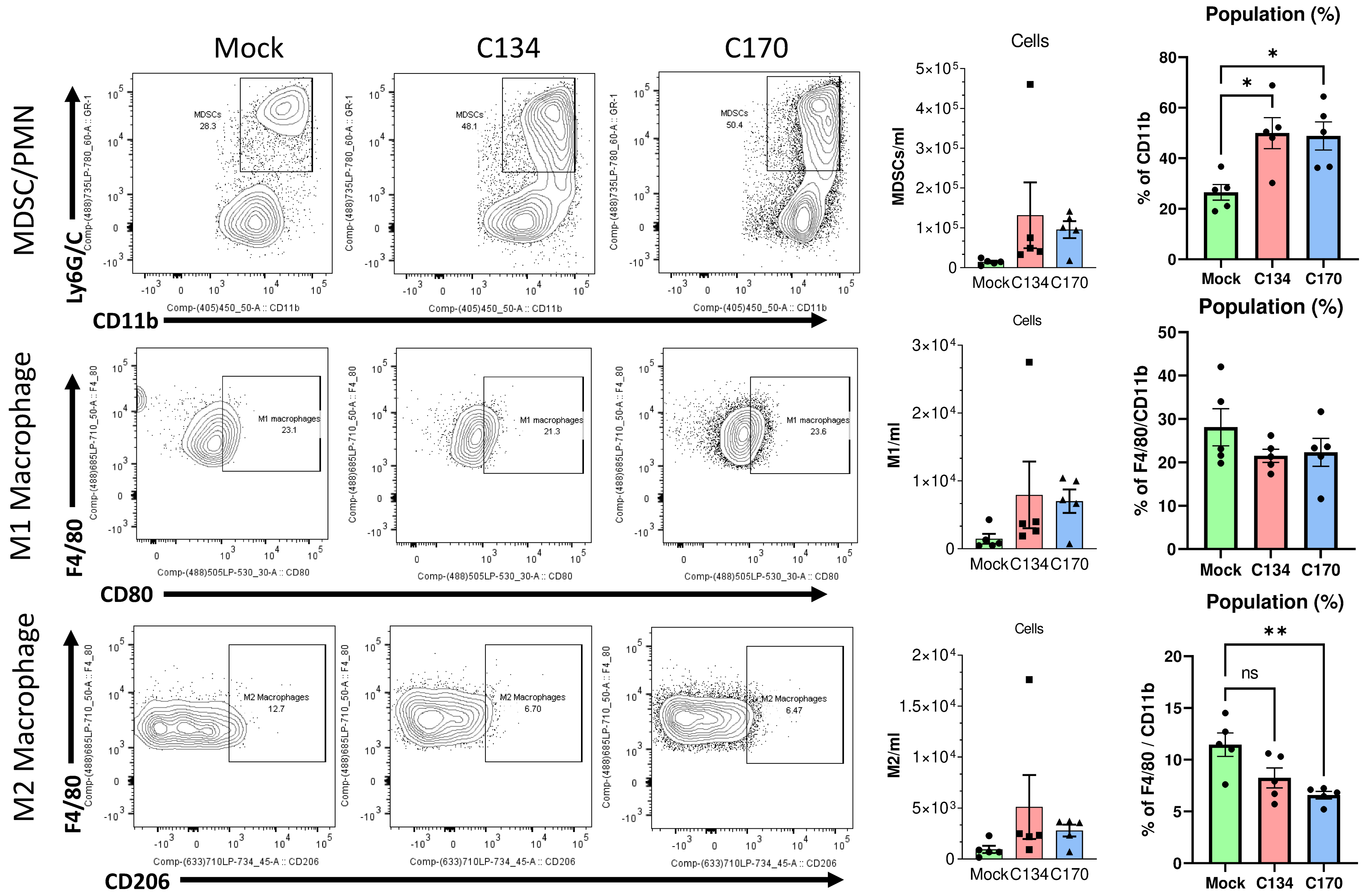

Supplemental Figure 3

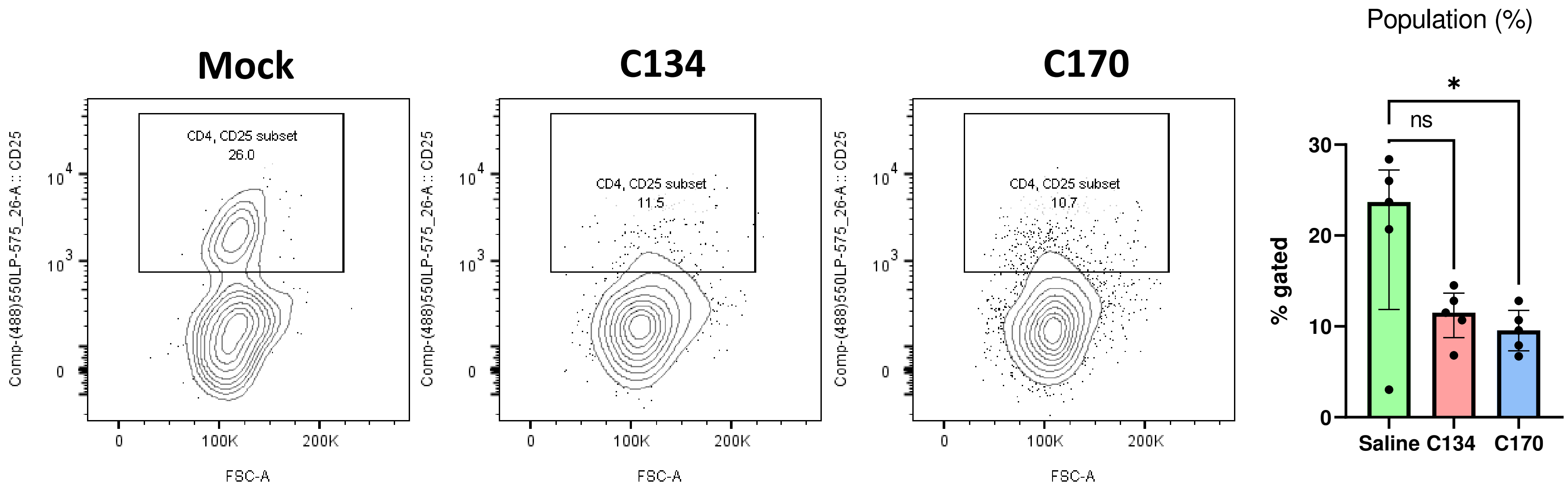

Supplemental Figure 4

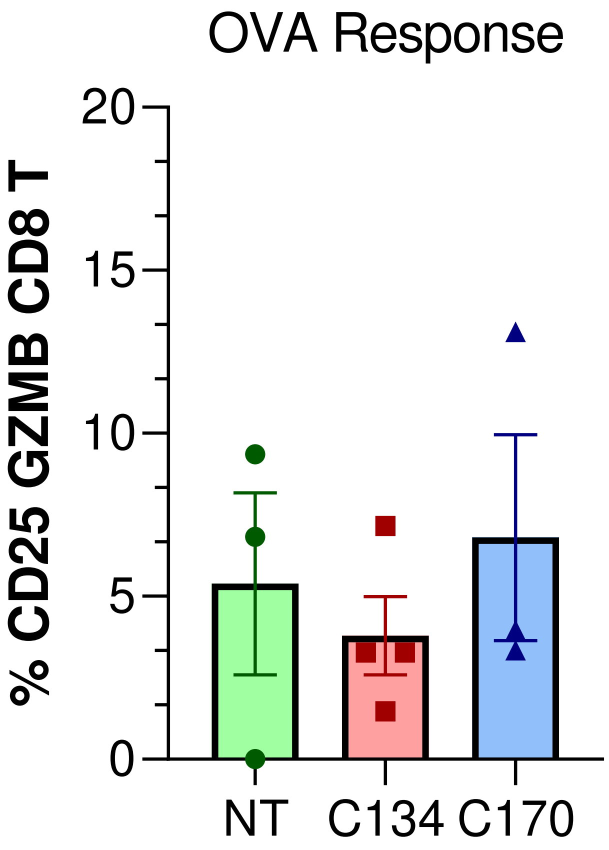

Supplemental Figure 5

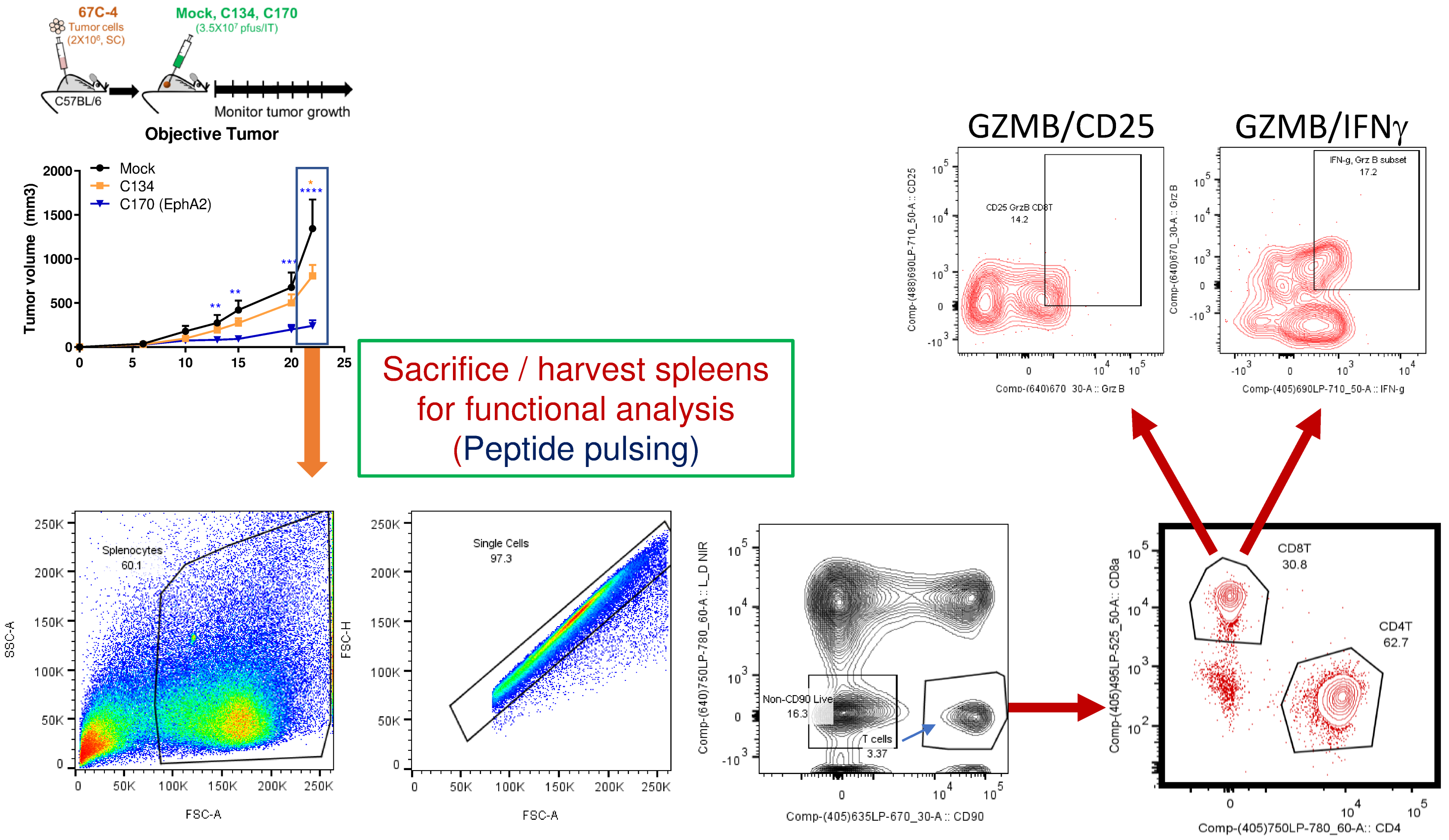

Supplemental Figure 6

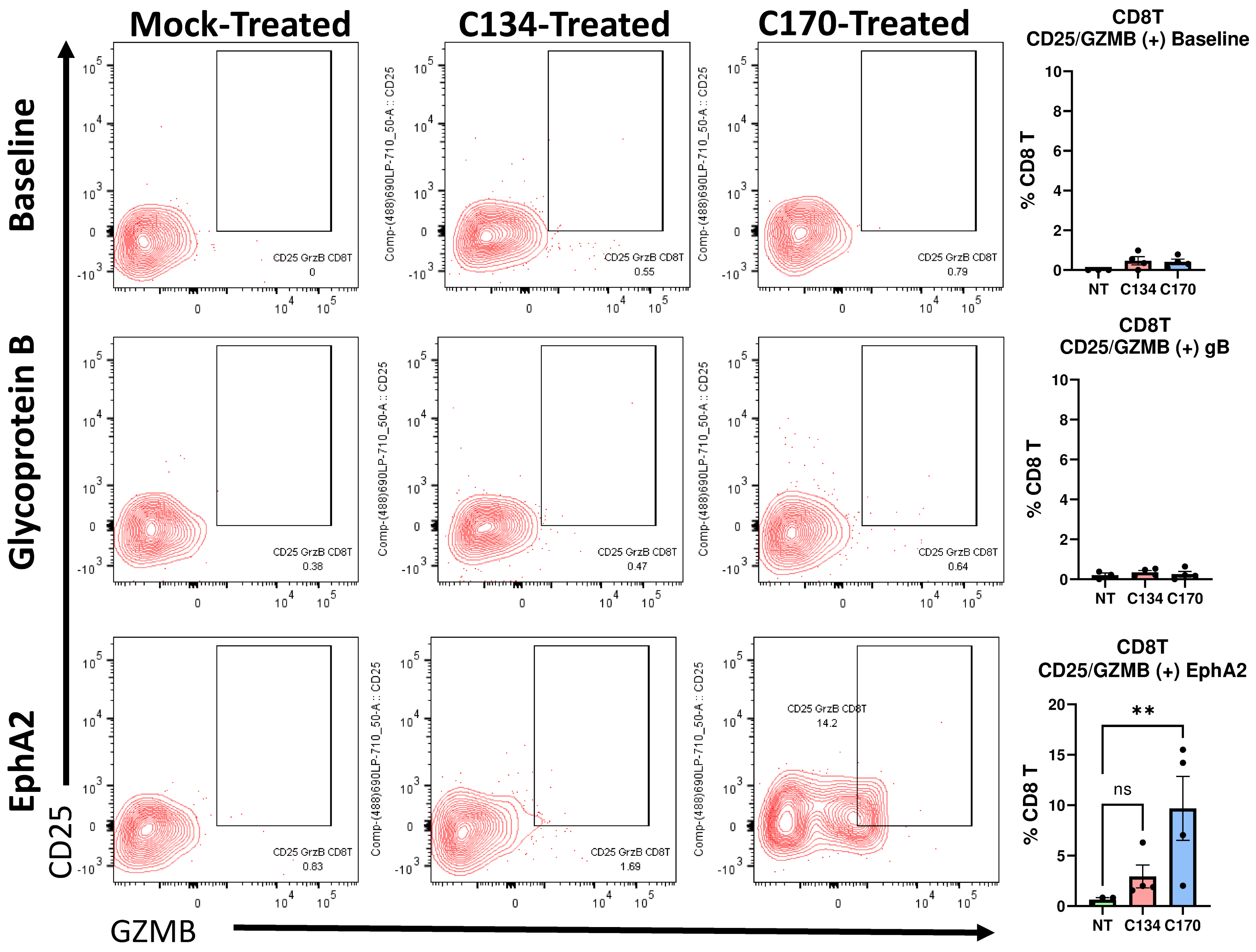

Supplemental Figure 7

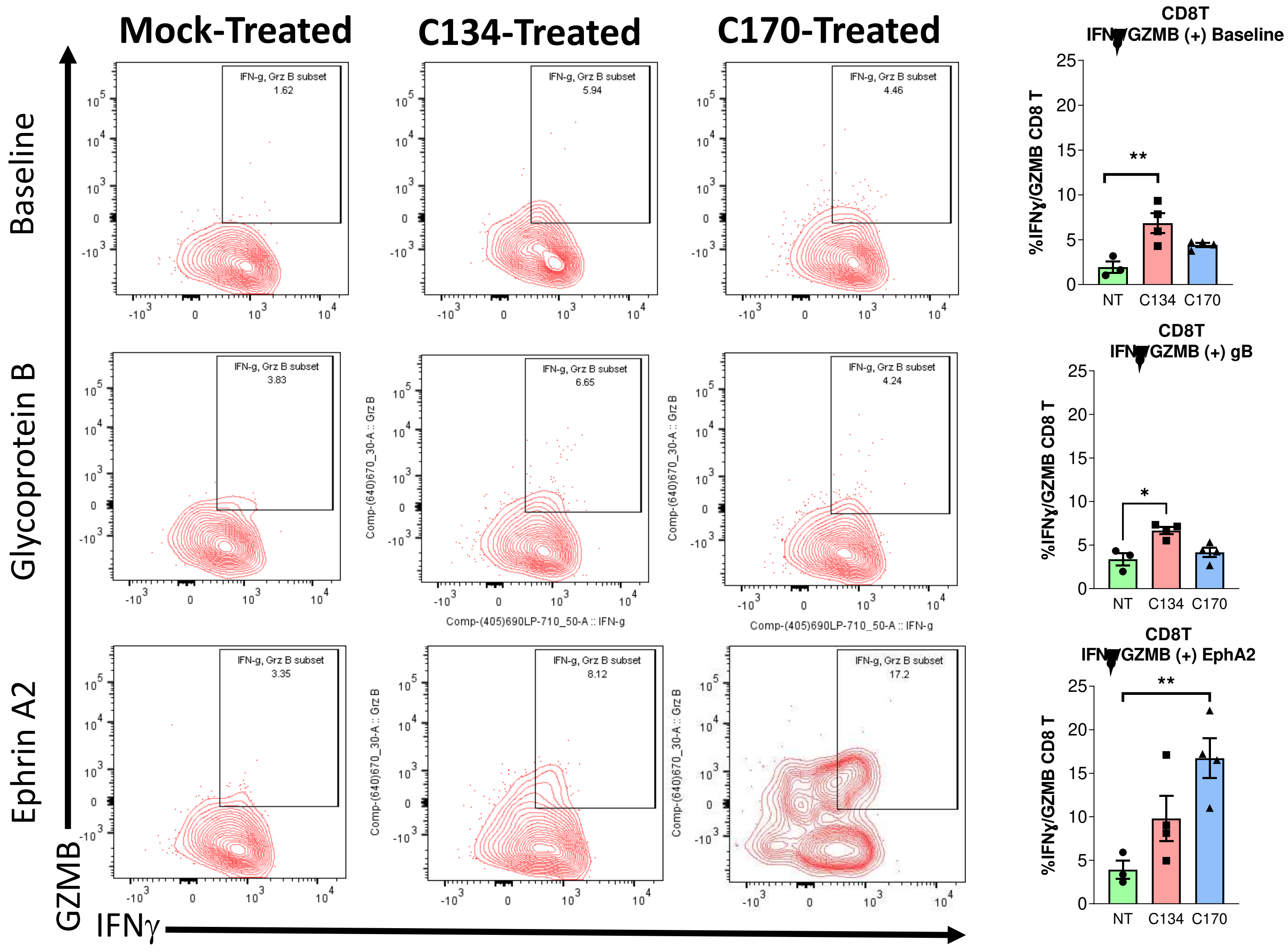

Supplemental Figure 8

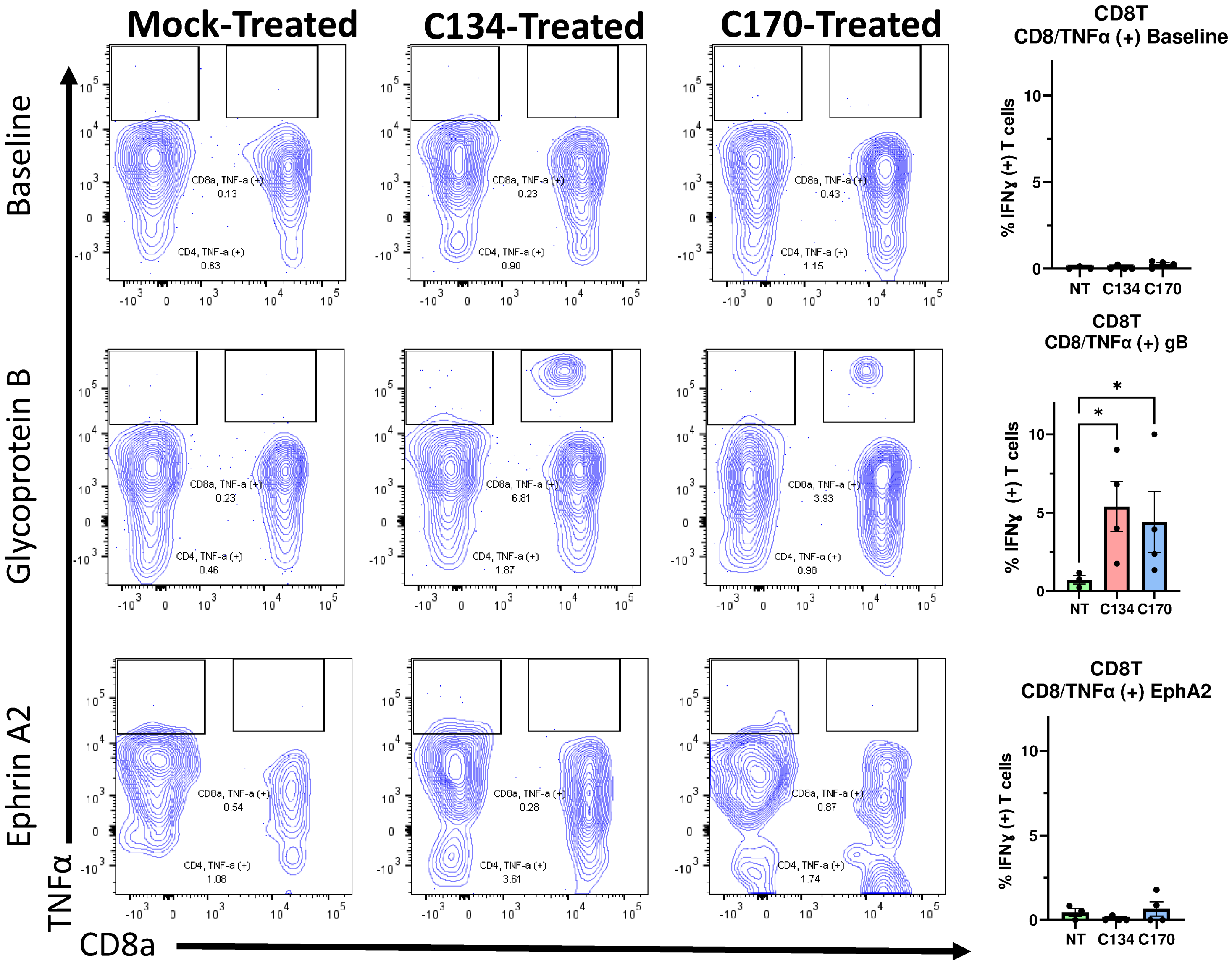

Supplemental Figure 9

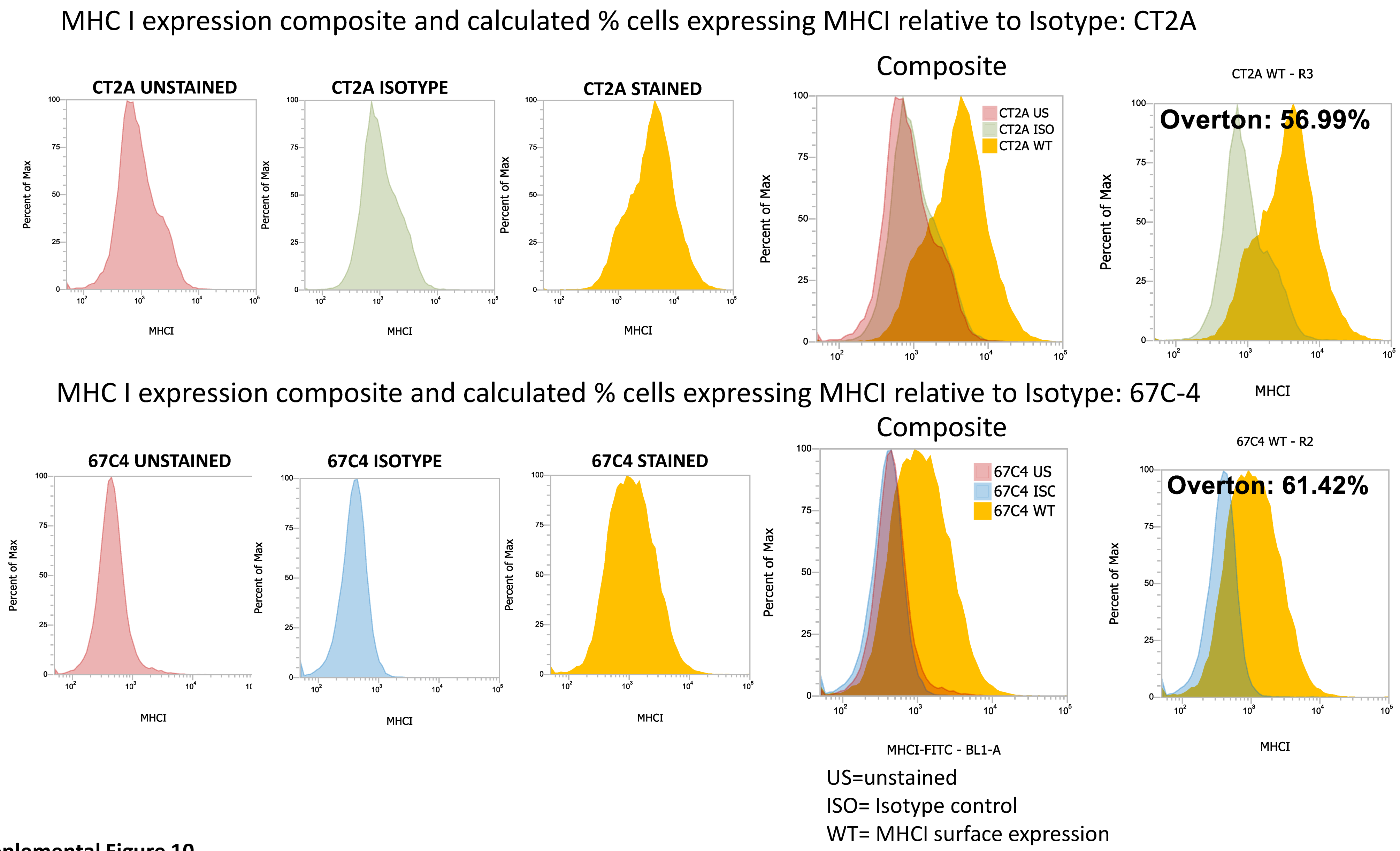

Supplemental Figure 10

EphA2 Protein Schematic

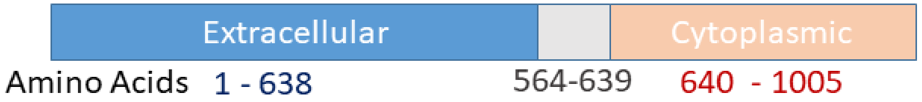

Pos	Peptide	B6 H2-Db (nM)	Rank	B6 H2-Kb (nM)	Rank
804	YGIVMWEVM	19.9	0.015	3661.6	5.5
926	FMVAGYTAI	61.7	0.04	1381.5	2.5
216	VAVSDTQPL	125.1	0.08	1829.5	3
498	LAPXDTTYL	134	0.08	7222.6	9.5
671	FSHHNIIRL	184.7	0.1	506.7	1.1
729	KYLANMNYV	394.9	0.175	3654.8	5.5
66	YSVCNVVSG	500.1	0.25	20500.6	28
331	SAPNYLTAI	705.1	0.3	76	0.25
845	SAIYQLXMM	1196.6	0.4	256.6	0.7
28	VVLLDFAAM	3680.3	1	108.6	0.3
601	AVLKFTTEI	4333.7	1.2	402.3	0.9
713	SVLQLVGML	5668.1	1.5	395.4	0.9
790	ISYRKFTSA	8660	2.5	11.5	0.04
726	SGMKYLANM	10668.7	3	11	0.03
4	RAVGFCLAL	15570.4	5	332.9	0.8
407	MNYTFAVEA	29797.9	14	281.6	0.7
558	FIHRRRRNL	36101.6	22	355.4	0.8
682	VVSKYKPMM	42639.8	38	395.1	0.9

Supplemental Figure 11
